# Microbial Diversity Analysis and Genome Sequencing Identify *Xanthomonas perforans* as the Pathogen of Bacterial Leaf Canker of Water Spinach (*Ipomoea aquatic*)

**DOI:** 10.3389/fmicb.2021.752760

**Published:** 2021-10-27

**Authors:** Ming Hu, Chuhao Li, Xiaofan Zhou, Yang Xue, Si Wang, Anqun Hu, Shanshan Chen, Xiuwen Mo, Jianuan Zhou

**Affiliations:** ^1^Guangdong Laboratory for Lingnan Modern Agriculture, Guangdong Province Key Laboratory of Microbial Signals and Disease Control, Integrative Microbiology Research Centre, South China Agricultural University, Guangzhou, China; ^2^Agricultural Technology Service Centre of Daojiao Town, Dongguan, China

**Keywords:** bacterial leaf canker of water spinach, 16S rDNA amplicon sequencing, MLSA analysis, pathogenicity tests, genome sequencing

## Abstract

*Ipomoea aquatica* is a leafy vegetable widely cultivated in tropical Asia, Africa, and Oceania. Bacterial leaf canker disease has been attacking the planting fields and seriously affecting the quality of *I. aquatica* in epidemic areas in China. This study examined the microbial composition of *I. aquatica* leaves with classical symptoms of spot disease. The results showed that *Xanthomonas* was overwhelmingly dominant in all four diseased leaf samples but rarely present in rhizospheric soil or irrigation water samples. In addition, *Pantoea* was also detected in two of the diseased leaf samples. Pathogen isolation, identification, and inoculation revealed that both *Xanthomonas* sp. TC2-1 and *P. ananatis* were pathogenic to the leaves of *I. aquatic*, causing crater-shaped ulcerative spots and yellowing with big brown rot lesions on leaves, respectively. We further sequenced the whole genome of strain TC2-1 and showed that it is a member of *X. perforans*. Overall, this study identified *X. perforans* as the causal pathogen of *I. aquatica* bacterial leaf canker, and *P. ananatis* as a companion pathogen causing yellowing and brown rot on leaves. The correct identification of the pathogens will provide important basis for future efforts to formulate targeted application strategy for bacterial disease control.

## Introduction

*Ipomoea aquatica* Forsk, usually called water spinach, water convolvulus, kangkong, and swamp cabbage, is native to East Asia and has been widely cultivated as a popular green leafy vegetable in tropical and subtropical regions (Ismail and Fun, 2003). In China, it is the only species of the genus *Ipomoea* that is grown in paddy fields. It can grow in all provinces of the Yangtze River Basin from April to October, and is suitable to grow in warm and humid climate, as well as fertile and wet soil. In addition to its high nutritive value to humans and animals, *I. aquatica* also possesses medicinal importance (Hu et al., 2010; Lawal et al., 2015, 2017). It can be taken orally to alleviate nervous debility, jaundice (Lawal et al., 2017), nosebleeds, and high blood pressure (Duke and Ayensu, 1985). The extract has been reported to inhibit prostaglandin synthesis (Tseng et al., 1992) and possess antidiabetic (Malalavidhane et al., 2000), antioxidant (Prasad et al., 2005b), antinematodal (Mackeen et al., 1997), and anticancer activities (Prasad et al., 2005a).

Water spinach is affected by a variety of foliar diseases, such as the white rust caused by *Albugo ipomoeae-aquaticae*, which happens in almost all the planting areas (Sawada, 1922; Safeeulla and Thrumalachar, 1953; Ho and Edie, 1969; Gao et al., 1985; Yu et al., 2015), the foliar diseases caused by *Phyllosticta ipomoeae*, *Cerocospora ipomoeae*, and *Pseudomonas syrigae* pv. *Syringae*, which were observed in commercial greenhouses in Ontario and California during the 1990s (Cerkauskas et al., 2006), spot blight caused by *Stagonosporopsis cucurbitacearum* in China (Liu et al., 2017), and leaf spot caused by *Myrothecium roridum* in Jianshui County, Yunnan Province, China, in May 2015 (Wang et al., 2017).

Bacterial leaf spot of water spinach was first observed in Bang Pai, Pasee Charoen, Bangkok, Thailand, in 1990, and the pathogen was initially identified as a pathovar of *Xanthomonas campestris* by biochemical characters and xanthomonadin pigment (Leksomboon et al., 1991). Records in the NCBI GenBank database indicate that the disease also happened in Fuzhou, Fujian Province, China, in 2015 where *X. perforans* was identified as the pathogen based on the *dnaK* (MN626337.1), *gyrB* (MN626338.1), *recA* (MN626339.1), and *rpoD* (MN626340.1) gene sequence similarities.

Since 2010, bacterial leaf canker (also called bacterial leaf spot) has occurred in Dongguan City, Guangdong Province, China. The scattered land scale of Dongguan City and the high cost of land rent bring a demand for the plantation of high-value economic crops. During the flood season from April to September every year, due to the continuous rainfall and the small land blocks among industrial and living regions, farmers are inclined to plant water spinach in the pattern of basically paddy field planting for its tolerance to waterlogging and high temperature. Farmers vividly called bacterial leaf canker “pockmarked disease” according to the crater shape around the leaf lesions. The disease is becoming increasingly serious and has significantly affected the production of water spinach, causing great economic losses to vegetable farmers. The pathogen of the disease in Dongguan City is unclear. Farmers mistakenly attributed the pockmarks to white rust and, therefore, often applied broad-spectrum protective fungicides such as mancozeb in the field, but received no controlling effect. Most farmers are helpless and have to temporarily give up field management and harvest until the weather becomes cool.

In the summer of 2019 and 2020, we surveyed the incidence of bacterial leaf canker of water spinach in Dongguan City and collected diseased leaves, water and soil samples for analysis of the microbial diversity using 16S rDNA amplicon sequencing. We also isolated and identified the pathogens using culture-based approaches. Results revealed that the pathogen responsible for the classical symptoms of pockmarked spots on leaves was *Xanthomonas* sp., and *Pantoea ananatis* was a companion bacterium causing yellowing with big brown rot lesions on leaves. Further identification of the *Xanthomonas* strain by genome sequencing revealed that it belongs to *X. perforans*.

## Materials and Methods

### Disease Investigation and Sample Collection

Dongguan City is located at 22°39′ to 23°09′ North latitude and 113°31′ to 114°15′ East longitude, in the south-central part of Guangdong Province, the East Bank of the Pearl River Estuary and the Pearl River Delta downstream of the Dongjiang River.

Since 2010, water spinach in towns and districts of Qingxi, Shatian, Chashan, Daojiao, and Wanjiang and other places in Dongguan City has suffered from a new disease (vividly called “pockmark” by farmers). In this study, the five sampling method was used to investigate the incidence rate of the disease in the summer of 2019 and 2020. Briefly, five points in each field and 50 clumps of water spinach at each point were examined. A total of 2,500 clumps in 10 fields were investigated. The incidence of disease was calculated as the percentage of infected clumps in total clumps surveyed. For the analysis of microbial diversity and pathogen isolation, samples from Wanjiang District were collected.

### Microbiota Analysis

Genomic DNA of symptomatic leaf (1 g), water (10 ml), and soils (5 g) were, respectively, extracted using ALFA-SEQ Advanced Plant DNA Kit (for the leaf) and ALFA-SEQ Advanced Soil DNA Kit (for water and soil samples) (Guangdong Magigene Biotechnology Co., Guangzhou, China) according to the manual protocols. Integrity, purity, and concentration of the DNA were examined using 1% agarose gel electrophoresis. The V3–V4 region of the 16S rDNA of the sample DNA was amplified using TaKaRa Premix Taq^®^ version 2.0 (TaKaRa Biotechnology Co., Dalian, China) with barcoded primers (338F 5′-ACTCCTACGGGAGGCAGCA-3′; 806R 5′-GGACTACHVGGGTWTCTAAT-3′) in conditions of pre-denaturation at 94°C for 5 min; 30 cycles of 94°C for 30 s, 52°C for 30 s, and 72°C for 30 s; extension at 72°C for 10 min on BioRad S1000 (Bio-Rad Laboratory, CA, United States). Three replicates were amplified for each sample and combined for further analysis. The amplicons were checked using 1% agarose gel electrophoresis, and fragments in size of 470 bp were purified using E.Z.N.A.^®^ Gel Extraction Kit (Omega, United States). A library was built according to the standard process of NEBNext^®^ Ultra^TM^ DNA Library Prep Kit for Illumina^®^ (New England Biolabs, United States). The products were sequenced by Illumina Nova 6000 PE250 technology at Guangdong Magigene Biotechnology Co., Guangzhou, China.

Paired-end clean reads were obtained after quality control by removing the primer sequences of the paired-end raw reads using the cutadapt software^1^ and assembled using arch-fastq_mergepairs V10 (parameters include the minimum overlap length as 16 bp and the maximum allowable mismatch of overlap region of splicing sequence as 5 bp)^2^ to obtain raw tags, which were subjected to quality tailoring (-W 4 -M 20) using fastp version 0.14.1^3^ to get the clean tags. The raw sequencing reads were deposited in the NCBI Sequence Read Archive database under the accession number: PRJNA739543. Operational taxonomic units (OTUs) were sorted and counted using the UPARSE software. Representative sequences of each OUT were annotated by the Ribosomal Database Project (RDP) to get the species sources. Community analysis and different abundance of OTUs were performed using STAMP 2.0.8^4^, vegan (R package).

### Microbial Isolation and Purification

Microbial separation from the symptomatic leaves was carried out using the method previously described (Li et al., 2020). First, diseased samples were cut into small pieces (0.5–1 cm^2^), and the surface was disinfected in sequence with 70% ethanol solution for 30 s, 5% sodium hypochlorite for 1 min, and sterile water washing for three times. Second, tissues were placed onto Luria–Bertani (LB, containing 10 g/L of typtone, 5 g/L of yeast extract, and 10 g/L of NaCl) agar (1.5% w/v) plates and potato dextrose agar (PDA, TOPBIO, Zhaoyuan, China) plates for incubation at 28°C for 24 h and 3 days, respectively. Colonies from the plates were, respectively, streaked onto fresh LB medium plates for purification and grown in LB liquid medium with shaking at 200 rpm overnight to preserve for further study.

### 16S rDNA Gene Sequencing of Single Colony

Bacteria were grown in LB medium until OD_600_ = 1.0, and genomic DNAs were extracted using the EasyPure Bacteria Genomic DNA Kit (TransGen Biotech, Beijing, China). The 16S rDNA gene sequences were amplified using the primers 27f and 1492r (Coenye et al., 1999) listed in Supplementary Table 1. The products were examined using 1% agarose gel electrophoresis and sent to Sangon Biotech Company in Shanghai, China, for sequencing. SeqMan V.5.00 was used to assemble sequences generated from forward and reverse primers.

### Pathogenicity Tests of the Isolated Strains

Single colonies of the tested strains were grown in 10 ml of LB medium at 28°C overnight and adjusted to OD_600_ = 1.5. Since the leaves of water spinach are very thin and tender, inoculation by acupuncture or injection *in vitro* or *in vivo* resulted in rapid rot (data not shown). Therefore, we have created a new inoculation method suitable for pathogenicity tests on tender leaves. Briefly, leaves with a similar size on the plants grown in a pot were chosen and wiped with 70% ethanol solution on the back. After drying, sterile toothbrushes dipped in each bacterial culture were used to brush gently on the lower side of three leaves. LB liquid medium was used as a negative control. The pots were kept in a growth chamber (Shanghai YiHeng Scientific Instruments Co., Ltd) with controlled conditions as 28 ± 2°C, 75 ± 15% relative humidity, and 12-h white light illuminance (7,350 lx). Photos were taken after 2- and 7-day post inoculation. The experiment was repeated twice.

To fulfill Koch’s postulates, the bacteria were re-isolated from the diseased leaves, and the 16S rDNA gene sequences were amplified and compared with the corresponding sequences of the inoculated ones.

For co-inoculation of TC2-1 and TC3-1, sterile toothbrushes were used to gently brush on the right half lower side of the blades to make microwounds, and 100 μl of bacterial cultures (grown in LB medium until OD_600_ = 1.5) of TC2-1, TC3-1, or TC2-1 + TC3-1 mixture was evenly spread onto the whole lower side of each blade. The pots were kept in the growth chamber. Photos were taken after 1- and 3-day post inoculation. The experiment was repeated three times.

To test the pathogenicity of *X. perforans* on different plants, the tested strains were grown in LB medium until OD_600_ = 1.0, and different plant organs were selected using different inoculation methods. For pepper (*Capsicum annuum*) and tomato (*Lycopersicon esculentum*) leaves, and mango (*Mangifera indica*) fruit, sterile toothbrushes dipped in TC2-1 culture were used to brush gently on the inoculated sites. For citrus (*Citrus reticulata*) leaves, TC2-1 was inoculated on the lower side by pressing a sterile puncher (5 mm) dipped with bacterial culture and incubated at 28°C. All trays were kept at 28°C until symptoms appeared. The same volume of LB medium was inoculated as a negative control. Each assay was repeated three times.

### Hypersensitive Response Assay

Bacteria were grown in LB medium until OD_600_ = 1.0 (greater than 10^9^ CFU/ml) (Kido et al., 2010). Leaves of *Nicotiana tabacum* variant K326 (Chen et al., 2018) were inoculated on the lower side by pressing a sterilized puncher (5 mm) dipped with bacterial culture and incubated at 28°C. A positive hypersensitive response (HR) reaction was recorded when the inoculated leaf tissue collapsed or light brown necrosis occurred within 48 h after inoculation (Lana et al., 2012). Each assay was repeated three times.

### Pathogen Identification by Multilocus Sequence Analysis

Pathogenic isolates that fulfilled Koch’s postulates were further identified using the multilocus sequence analysis (MLSA). For the *Xanthomonas* isolates, the *atpD* (ATP synthase β subunit) (Brady et al., 2008), *avrBs2* (Type III secretion effector AvrBs2) (Hajri et al., 2009), *gyrB* (DNA gyrase subunit B) (Parkinson et al., 2007), *cpn60* (60-kDa chaperonin protein subunits) (Hill et al., 2004), and *rpoD* (RNA polymerase sigma factor) (Tian, 2018) genes were selected for PCR amplification; for the *Pantoea* isolates, the *atpD*, *gyrB*, *infB* (initiation translation factor 2), and *rpoB* (DNA-directed RNA polymerase subunit beta) (Brady et al., 2008) gene sequences were, respectively, amplified using the primers listed in Supplementary Table 1. Amplicons were purified with a PCR Purification Kit (TransGen Biotech, Beijing, China) and sequenced by Sangon Biotech Company in Shanghai, China. Sequences were submitted to the GenBank database with accession nos. indicated in Supplementary Table 2.

In view of the classification confusion and evolution inconsistency of different housekeeping genes in *Xanthomonas* genus, phylogenetic trees of isolates TC1-1 and TC2-1 were built based on each gene sequence to reveal their taxonomic status; for isolates TC3-1 and TC3-2, phylogenetic analysis was performed based on the joint sequences of *atpD*, *gyrB*, *infB*, and *rpoB* genes of the two isolates and their corresponding close-related strains obtained from NCBI database. Sequences were aligned with ClustalW, and trees were constructed using the MEGA 6.0 software by the maximum-likelihood method with 1,000 bootstrap replicates.

### Genome Sequencing, Assembly, and Annotation

Genomic DNA was extracted from TC2-1 in LB medium culture using the SDS method. The harvested DNA was detected by the agarose gel electrophoresis and quantified by Qubit^®^ 2.0 Fluorometer (Thermo Fisher Scientific, Waltham, MA, United States). The complete genome was sequenced by Novogene (Tianjin, China) using the Nanopore PromethION platform and Illumina NovaSeq platform. The sequence was first assembled with long read data and then polished with both long and short read data by medaka v1.2.5 and unicycler v0.4.8 (Wick et al., 2017), respectively. The sequencing data and genome assembly have been deposited in the NCBI database under the accession no. PRJNA742079.

PGAP (2021-01-11.build5132) (Tatusova et al., 2016) was used to predict gene structure and gene function of the complete genome. Islandviewer4 (Bertelli et al., 2017) was used to predict genomic islands. Phaster (Arndt et al., 2016) was used to predict prophage. TXSScan was used to annotate secretion systems (Abby and Rocha, 2017). Effectors were predicted by aligning all protein sequence of TC2-1 using BLASTP with validated effectors in the T3SE (Hu et al., 2017), T4SE (Bi et al., 2013; Zou et al., 2013; An et al., 2018), and T6SE databases (Li et al., 2015; An et al., 2018). Hits with e-value less than 1e^–5^ and qcov_hsp_perc more than 50 were identified as effectors.

### Phylogenetic Analysis of TC2-1 and Genomic Comparison of Available Genomes of *Xanthomonas perforans* Strains

Phylogenetic analysis was performed by GTDB-tk v1.4.0 (Chaumeil et al., 2020). In detail, TC2-1 was placed to a pre-built bacteria-wide phylogenetic tree according to the sequence identity of 120 single-copy conserved genes, and then ANI values between TC2-1 and its closely related representative strains on the tree was calculated by fastANI. The taxonomic identity of TC2-1 was determined based on both the phylogenetic and ANI analyses. A separate phylogenetic tree including TC2-1 representative *Xanthomonas* strains was extract from the output of GTDB-tk, and the ANI value between strains was calculated again by the fastANI. Tree files were operated by ete3 (Huerta-Cepas et al., 2016), and phylogenetic tree was visualized by iTOL (Letunic and Bork, 2019).

To identify TC2-1-specific genes, a phylogenetic tree of TC2-1 and 141 *X. perforans* retrieved from the NCBI RefSeq database was built by the method described previously (Pierre-Alain et al., 2018). Then TC2-1 and five representative strains were selected based on the phylogenetic tree, namely, *X. perforans* NI1 (GCF_003136155.1), *X. perforans* Xp11-2 (GCF_001009445.1), *X. perforans* AL66 (GCF_007714065.1), *X. perforans* GEV2392 (GCF_006979735.1), and *X. perforans* CFBP 7293 (GCF_001976075.1), which were analyzed by orthofinder v2.5.2 (Emms and Kelly, 2015). TC2-1-specific genes were then screened based on the orthofinder results after filtering the protein sequences according to the parameters (qcovhsps > 80% and identity > 65%) compared with those of *X. perforans* in the NCBI database.

## Results

### Disease Incidence and Symptoms of Bacterial Leaf Canker of Water Spinach in Dongguan City

Since 2010, bacterial leaf canker disease has attacked water spinach cultivated in many towns and districts of Dongguan City, Guangdong Province, China. Due to scattered field planting, it is difficult to calculate the total planting area of water spinach. Therefore, we investigated some cultivated fields in Daojiao Town, Gaobu Town, and Wanjiang District in the summer of 2019 and 2020. The occurrences of the disease on “Baijun 311” and “Baijun 611” cultivars are 100% in all the surveyed places, while the occurrences on “Qingtong 311” cultivar are 16.7, 86, and 100% in Daojiao, Wanjiang, and Gaobu, respectively (Supplementary Table 3).

**FIGURE 1:**
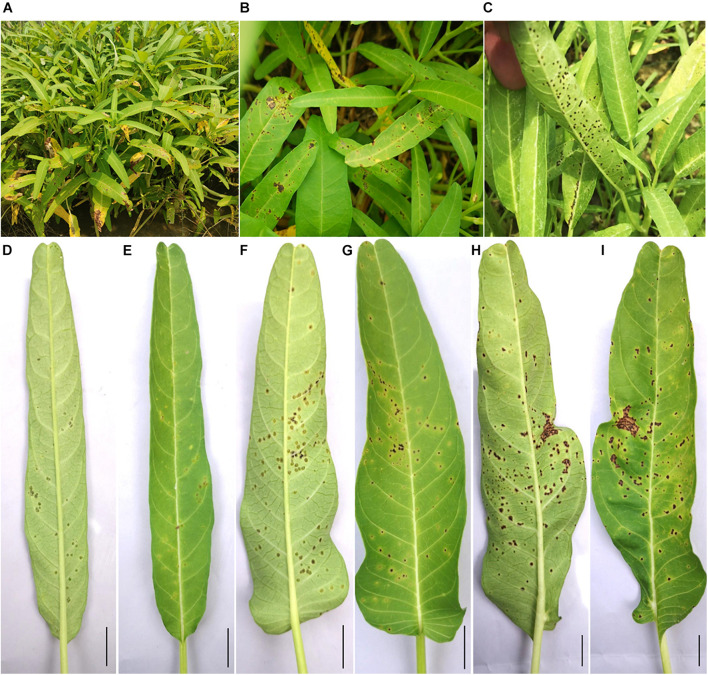
Symptoms of bacterial leaf canker of water spinach in the field. **(A–C)** Diseased water spinach plants in the field. **(D,E)** Early symptoms on the lower and upper side of the leaf. **(F,G)** Leaf symptoms in the following course of disease development on the lower and upper side of leaf. **(H,I)** Leaf symptoms in the later stage on the lower and upper side of the leaf. Scale bar = 1 cm.

When the disease occurs, some leaves become yellow (Figure 1A), while some remain green, with brown or chlorotic spots observed on both (Figure 1B). Small raised black spots are observed on the lower side of the leaf (Figure 1C). In the early phase of infection, water-soaked transparent spots emerge on the lower side of the leaves, protuberant, edge clear (Figure 1D). Chlorotic spots appear on the upper side of the leaves, flat, edge dim, and irregular, and the middle of the lesions gradually turns brown (Figure 1E). In the following course of disease development, more and more watery spots grow on the lower side of the leaves, gradually expand, and turn brown, with a yellow halo on the edge (Figure 1F). Chlorotic spots turn larger on the leaf surface with brown in the middle and irregular yellow halo on the edge (Figure 1G). Finally, some disease spots expand and become connected; some leaves curl up and deform; the edge of the spots protrudes into crater -like cankers on the lower side (Figure 1H), which appear as brown necrosis with a yellow halo around on the upper side (Figure 1I). During the whole period of the disease, the roots and the stems remain healthy, indicating that the pathogens preferentially colonize mesophyll tissue.

### Taxa Abundance and Diversity of the Samples

Leaves and rhizospheric soil of diseased plants and irrigation water near the planting field were collected from Dafen Zone, Wanjiang District, and the microbiomes were determined by 16S rDNA amplicon sequencing. A total of about 84,000 raw paired-end reads were generated for each sample (Supplementary Table 4). The proportions of high-quality reads were between 3.23 and 25.74% for leaf samples, between 49.38 and 55.40% for soil samples, and as high as 84.83% for the water sample (Supplementary Table 4 and Supplementary Figure 1). A combined analysis of high-quality reads from all samples identified a total of 4,903 OTUs, among which 333 were detected in the four leaf tissues, 1,100 were detected in the water sample, and 4,268 were detected in the three soil samples.

**FIGURE 2:**
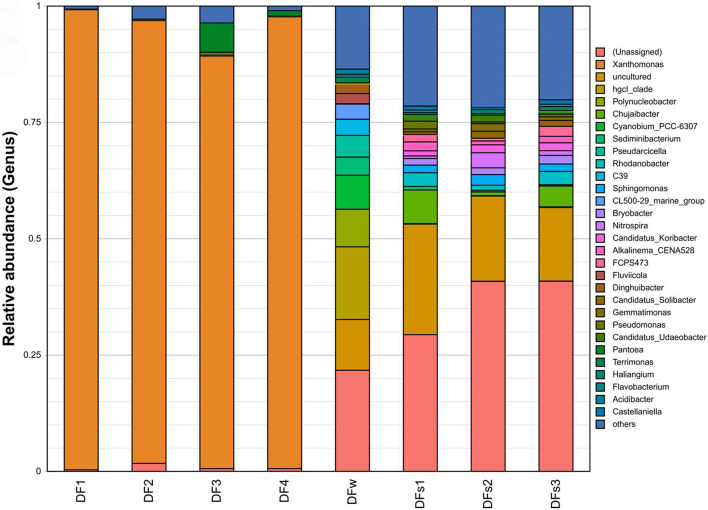
Relative abundance of the top 30 16S rDNA-based operational taxonomic units (OUTs) at the genus level. Samples are labeled to indicate their source of collection: DF1 to DF4 indicate four leaf samples of diseased plants from Dafen Zone. DFs1 to DFs3 indicate three rhizosphere soil samples of diseased plants from Dafen Zone. DFw indicates nearby irrigation water of diseased plants from Dafen Zone.

The most abundant OTUs are vastly different among samples (Supplementary Table 5). In leaf samples, *Xanthomonas* was the genus with the highest relative abundance in the community (ranging from 88.64 to 98.88%), and *Pantoea* ranked second in two of the samples. In contrast, in the water and soil samples, the proportion of *Xanthomonas* was between 0.06 and 0.39%, and the proportion of *Pantoea* was between 0 and 0.15% (Figure 2 and Supplementary Table 5).

### Molecular Identification of Bacterial Strains

Diseased leaf samples collected from Wanjiang District were subjected to pathogen isolation. As a result, a total of 23 isolates were obtained from the leaf tissues, including 5 *Xanthomonas* sp. isolates (TC1-1, TC1-2, TC2-1, TC2-3, and TCX21), 3 *Pantoea* sp. isolates (TC3-1, TC3-2, and TC3-5), 1 *Pseudomonas aeruginosa* isolate (TC11), and 3 additional *Pseudomonas* sp. isolates (TC1-3, TC3-3, and TC3-4), as well as 1 *Bacillus aquimaris* isolate (TC1-4) and 10 additional *Bacillus* spp. isolates (TC2-2, TC2-4, TCX22, TCX31, TCX32, TCX33, WJ2-1, WJ1, WJ2-2, and WJ3) (Supplementary Table 6). For *Xanthomonas* isolates, all the 16S rDNA gene sequences are completely identical to those of *X. axonopodis* pv. *commiphoreae* strain LMG26789, *X. perforans* strains 91-118 and LH3, and 99.93% identical to that of *X. axonopodis* pv. *citrumelo* F1. For three *Pantoea* isolates, the 16S rDNA gene sequences are completely identical to that of *Pantoea ananatis* SGAir0210, and 99.73% are identical to that of *P. ananatis* PP1.

**FIGURE 3:**
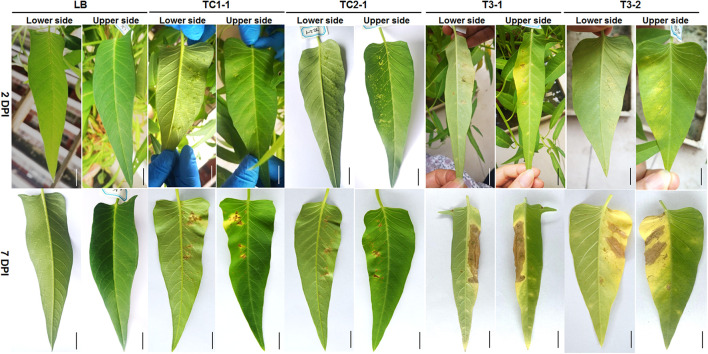
Symptoms of water spinach after inoculation with LB, strains TC1-1, TC2-1, TC3-1, and TC3-2, respectively. Photos were taken after 2 and 7 DPI (days post inoculation), respectively. Scale bar = 1 cm.

To determine which isolates are pathogenic, we performed pathogenicity tests of TC1-1, TC2-1, TC1-3, TC1-4, TCX11, WJ2-1, TC3-1, TC3-2, WJ1, WJ2-2, and WJ3 on leaves of water spinach, and found that only isolates TC1-1, TC2-1, TC3-1, and TC3-2 are pathogenic (Figure 3). Two days after inoculation, dense water-soaked spots raised from the back of the leaves inoculated with *Xanthomonas* isolates TC1-1 and TC2-1, and the spots are chlorotic and yellow with clear edge on the leaf surface (Figure 3). On the other hand, small chlorotic spots appeared on the back of the leaves inoculated with *Pantoea* isolates TC3-1 and TC3-2; lesions were yellow, irregular, and indistinct with non-swollen mesophyll tissue on the leaf surface (Figure 3). At 7 days post inoculation, the protuberant spots in the TC1-1- and TC2-1-inoculated groups turned brown, and some expanded together with a yellow halo around the lesions. In general, the number of spots did not increase significantly (Figure 3). In comparison, in the TC3-1-and TC3-2-inoculated groups, the disease spots rapidly spread and connected into blocks with mesophyll tissue rotted and turned brown. The leaves were seriously yellowing on the inoculated site (Figure 3). According to the disease symptoms of the inoculated leaves, those inoculated with isolates TC1-1 and TC2-1 were similar to the typical symptoms of the bacterial canker disease. Thus, we observed *Xanthomonas* isolates TC1-1 and TC2-1 as the pathogens of the bacterial leaf canker of water spinach.

**FIGURE 4:**
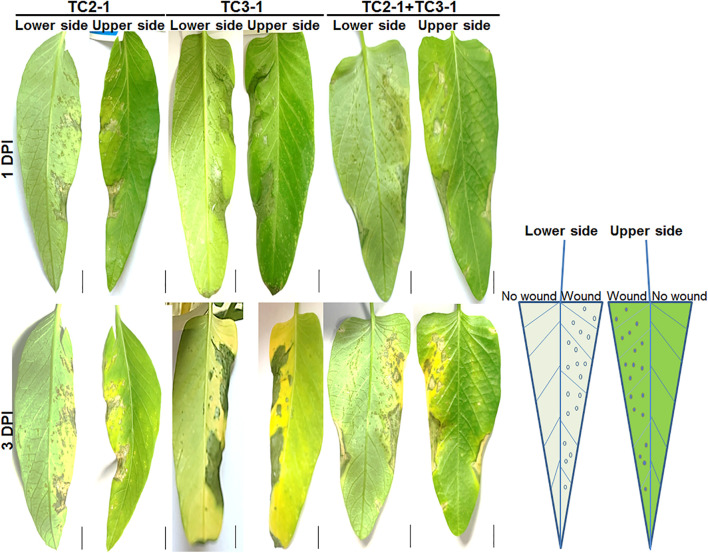
Co-inoculation of TC2-1 and TC3-1 on water spinach leaves. Sterile toothbrushes were used to gently brush on the right half lower side of blades to make micro wounds, 100 μl of bacterial cultures (grown in LB medium until OD_600_ = 1.5) of TC2-1, TC3-1, or TC2-1 + TC3-1 mixture were evenly spread onto the whole lower side of each blade. Photos were taken after 1 and 3 DPI (days post inoculation), respectively. Scale bar = 1 cm.

The simultaneous isolation of both *Pantoea* and *Xanthomonas* from diseased plants raises the possibility of synergistic or antagonistic interaction between both pathogens. Thus, co-inoculation of TC2-1 and TC3-1 were performed, and results showed that in the condition of wound pre-treatment, the co-inoculation of both pathogens led to similar symptoms as the inoculation of TC2-1 alone. However, for non-wound pre-treatment, which resemble the condition of natural infection, co-inoculation of both pathogens resulted in accelerated disease development and more severe symptoms compared with the inoculation of either pathogen individually (Figure 4).

To refine the taxonomic position of the pathogenic isolates, we performed MLSA analysis based on five housekeeping genes (*atpD*, *avrBs2*, *cpn60*, *gyrB*, and *rpoD*) for isolates TC1-1 and TC2-1, and four housekeeping genes (*atpD*, *gyrB*, *infB*, and *rpoB*) for isolates TC3-1 and TC3-2. As a result, the single-gene phylogenies of *atpD*, *cpn60*, *gyrB*, and *rpoD*, as well as the joint phylogenetic tree of all five genes, all contain a monophyletic clade consisting of TC1-1, TC2-1, and six other strains including *X. axonopodis* pv. *citrumelo* F1, *X. axonopodis* pv. *commiphoreae* strain LMG26789, *X. perforans* strains 91-118 and LH3, *X. euvesicatoria* LMG930, and *X. campestris* pv. *vesicatirua* 85-10 (Supplementary Figure 2). However, the phylogenetic results were inconclusive on the closest relatives of TC1-1 and TC2-1, leaving the taxonomic status of both strains at the species level uncertain. A high similarity between strains in different species indicates inconsistency in the current *Xanthomonas* classification situation. Therefore, we decided to sequence the whole genome of one of the isolates TC2-1 to determine the exact taxonomy of bacterial leaf canker pathogen of water spinach.

In addition, TC3-1 and TC3-2 have completely identical sequences of *atpD*, *gyrB*, *infB*, and *rpoB*, which are most similar to those of strain SGAir0210, sharing 99.11, 99.18, 99.38, and 99.36% identities, respectively. Joint phylogenetic analysis of *atpD*, *gyrB*, *infB*, and *rpoB* also indicated that strains TC3-1 and TC3-2 are grouped together with *P. ananatis* strains (Supplementary Figure 3). Thus, based on these results, we can conclude that strains TC3-1 and TC3-2 belong to *Pantoea ananatis*.

### Species Classification of Isolate TC2-1 Based on Its Complete Genome Information

**FIGURE 5:**
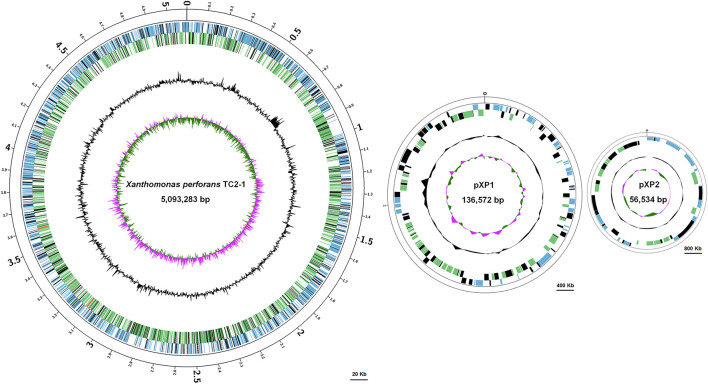
Circular map of the chromosome and two plasmids pXP1 and pXP2 of *Xanthomonas perforans s*train TC2-1. The circles from outside to inside represent feature of the positive strand, CDS (blue), rRNA (red), pseudogene (black); feature of the negative strand, CDS (green), rRNA (red), pseudogene (black); GC content; and GC-skew. Bars were indicated under each of the circular maps.

The TC2-1 genome was sequenced by a combination of Illumina short-read and Nanopore long-read sequencing technologies, and assembled into one circular chromosome (size: 5,093,283 bp; GC content: 64.76%) and two plasmids (sizes: 136,572 and 46,534 bp; GC contents: 60.96 and 62.10%) (Figure 5 and Table 1). A total of 3,848 protein-coding genes, 90 RNA genes (6 rRNAs, 51 tRNAs, and 30 ncRNAs), 671 pseudogenes, and 334 transposases (some are predicted as CDSs, some are pseudogenes) are annotated in the TC2-1 genome (GenBank no. CP077785.1) (Table 1).

**TABLE 1:** Genomic features of *Xanthomonas perforans* TC2-1.

Features	Chromosome	pXP1	pXP2	Total
Size (bp)	5,093,283	136,572	46,534	5,276,389
GC content (%)	64.76	60.96	62.10	64.64
Gene	3,820	81	37	3,938
CDS	3,730	81	37	3,848
RNA genes	90	0	0	90
rRNA	6	0	0	6
tRNA	51	0	0	51
ncRNA	30	0	0	30
RNase_P_RNA	1	0	0	1
SRP_RNA	1	0	0	1
Pseudogene	599	60	12	671
Riboswitch	6	0	0	6
tmRNA	1	0	0	1
Transposase	289	40	5	334

**FIGURE 6:**
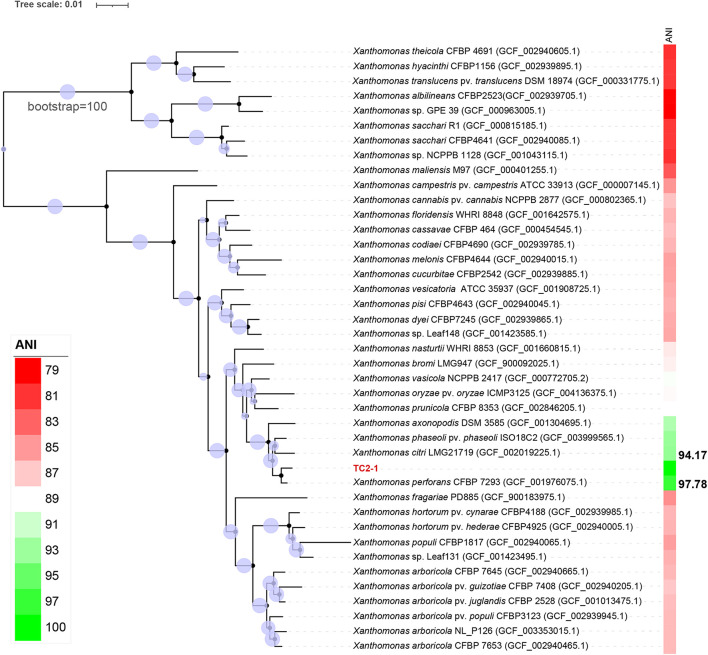
Phylogenetic tree of strain TC2-1 and the representative strains in genus *Xanthomonas* listed in the GTDB. ANI analysis was used by means of GTDB-Tk (Chaumeil et al., 2020). The closest strain of TC2-1 is *X. perforans* CFBP 7293 with a 97.78% ANI value.

The genome sequence of TC2-1 was analyzed with GTDB-Tk, which first placed TC2-1 in a pre-built bacterial phylogeny based on 120 proteins and then calculated the average nucleotide identity (ANI) values between TC2-1 and closely related type strains on the tree (Chaumeil et al., 2020). The result showed that TC2-1 is a member of *X. perforans*; the ANI value between TC2-1 and the type strain of *X. perforans* (strain CFBP 7293) is 97.78%, which is above the 95% threshold commonly used for bacterial species classification (Jain et al., 2018; Figure 6 and Supplementary Table 7). Additionally, the ANI values between TC2-1 and all 141 *X. perforans* genomes in the NCBI RefSeq database are above 97% (Supplementary Figure 4). Therefore, the water spinach pathogenic strain TC2-1 belongs to *Xanthomonas perforans*. To the best of our knowledge, water spinach is the fourth reported natural host plant of *X. perforans* besides tomato, pepper, and *Eucalyptus pellita*.

### Genome Comparison Between Strain TC2-1 and Other *Xanthomonas perforans* Strains

To identify TC2-1-specific genes related to host specialization, we performed a phylogenetic analysis of TC2-1 and all the 141 sequenced *X. perforans* strains in the NCBI RefSeq database based on 120 conserved, single-copy genes (Supplementary Figure 4) and selected five representative strains for further comparative analysis, including four strains isolated from tomato leaves (strain NI1 from Northern guinea savannah, and strains Xp11-2, GEV2392, and CFBP 7293 from different regions of the US) and one (strain AL66) isolated from pepper. Proteins present only in TC2-1, but not the other five strains, were then searched against the NCBI NR database to remove those with matches of high coverage (>80%) and identity (>65%) in any *X. perforans* strains. As a result, we identified a total of 277 genes that are unique to TC2-1, encoding 121 hypothetical proteins, 101 transposases, and 7 transcriptional regulators (Supplementary Table 8).

It is generally believed that there is a correlation between the repertoire of T3SEs and the host specificity of pathogenic *Xanthomonas* strains (Hajri et al., 2009, 2012a,b; Jacques et al., 2016). In the TC2-1 genome, we found an intact Type-III secretion system and predicted 56 T3SEs that are homologous to validated T3SEs in *Xanthomonas* and other bacteria (Table 2). Notably, 15 of these predicted T3SEs share low levels of sequence similarity with known T3SEs and, thus, were classified as TC2-1 unique. Among the predicted T3SEs, 19 encode short fragments of XOO4824 or XopT (Table 2), sharing high identity to each other; most of them were found to be adjacent to transposable elements, resulting in multiple copies in the genome. Actually, the real XopT and XOO4824 homologs in TC2-1 are TC2-1_004427 and TC2-1_004580, respectively, with 98.80% identity to each other. In summary, the number of predicted intact T3SEs in *X. perforans* strain TC2-1 is 37 indeed.

**TABLE 2:** Homolog of T3SE genes in *X. perforans* TC2-1.

Gene loci	Protein	Organism of the validated protein	Sequence similarity (%)
TC2-1_000053	AvrBs2	*Xanthomonas oryzae* pv. *oryzae* KACC10331	89.747
TC2-1_000255	XopR	*Xanthomonas campestris* pv. *vesicatoria* 85-10	99.476
TC2-1_000349	HrpF	*Xanthomonas campestris* pv. *vesicatoria* 85-10	98.025
TC2-1_000355	HrpE	*Xanthomonas campestris* pv. *vesicatoria* 85-10	77.419
TC2-1_000366	HrpB2	*Xanthomonas campestris* pv. *vesicatoria* 85-10	99.231
TC2-1_000381	XopA	*Xanthomonas campestris* pv. *vesicatoria* 85-10	97.436
TC2-1_000382	BapC	*Burkholderia pseudomallei* K96243	44.355
TC2-1_000383	XopM	*Xanthomonas campestris* pv. *vesicatoria* 85-10	98.266
TC2-1_000509	XopX	*Xanthomonas oryzae* pv. *oryzae* KACC10331	59.556
TC2-1_000682	XopV	*Xanthomonas campestris* pv. *vesicatoria* 85-10	94.207
TC2-1_001172	Cpn0490	*Chlamydophila pneumoniae* CWL029	30.405
TC2-1_001178	RipTPS	*Ralstonia solanacearum* GMI1000	40.839
TC2-1_001236	CT610	*Chlamydia trachomatis* D/UW-3/CX	23.077
TC2-1_001263	XAC3090	*Xanthomonas axonopodis* pv. *citri* str. 306	81.559
TC2-1_001267	XopK	*Xanthomonas campestris* pv. *vesicatoria* 85-10	98.118
TC2-1_001324	Mlr6361	*Mesorhizobium loti* MAFF303099	39.568
TC2-1_001337	AvrBsT	*Xanthomonas euvesicatoria* Bv5-4a	23.427
TC2-1_001346	RipAL	*Ralstonia solanacearum* UW551	46.779
TC2-1_001847	RipAP	*Ralstonia solanacearum* UW551	29.338
TC2-1_002077	XopZ	*Xanthomonas fuscans* subsp. *aurantifolii* str. ICPB 10535	91.354
TC2-1_002411	Pgl	*Bradyrhizobium diazoefficiens* USDA 110	38.235
TC2-1_002958	XopF2	*Xanthomonas campestris* pv. *vesicatoria* 85-10	99.279
TC2-1_002961	XopN	*Xanthomonas oryzae* pv. *oryzae* KACC10331	63.15
TC2-1_003035	T3SE	*Xanthomonas campestris* pv. *badrii* NEB122	90.72
TC2-1_003236	RipC1	*Ralstonia solanacearum* GMI1000	47.291
TC2-1_003276	SrfJ	*Salmonella enterica* subsp. *enterica* serovar Typhimurium str. SL1344	33.556
TC2-1_003677	HopAK1	*Pseudomonas syringae* pv. *tomato* str. DC3000	23.333
TC2-1_003780	HopK1	*Pseudomonas syringae* pv. *tomato* str. DC3000	37.681
TC2-1_003954	HopG1	*Pseudomonas syringae* pv. *tomato* str. DC3000	47.216
TC2-1_004171	AvrXacE1	*Xanthomonas axonopodis* pv. *citri* str. 306	92.269
TC2-1_004204	XopS	*Xanthomonas campestris* pv. *vesicatoria* 85-10	95.548
TC2-1_004427	XopT	*Xanthomonas fragariae* NBC2815	90.78
TC2-1_004430	XOO4824	*Xanthomonas oryzae* pv. *oryzae* KACC10331	65.672
TC2-1_004432	XOO4824	*Xanthomonas oryzae* pv. *oryzae* KACC10331	67.164
TC2-1_004435	XOO4824	*Xanthomonas oryzae* pv. *oryzae* KACC10331	67.164
TC2-1_004439	XOO4824	*Xanthomonas oryzae* pv. *oryzae* KACC10331	68.657
TC2-1_004442	XOO4824	*Xanthomonas oryzae* pv. *oryzae* KACC10331	65.672
TC2-1_004445	XOO4824	*Xanthomonas oryzae* pv. *oryzae* KACC10331	69.231
TC2-1_004449	XOO4824	*Xanthomonas oryzae* pv. *oryzae* KACC10331	68.657
TC2-1_004455	XOO4824	*Xanthomonas oryzae* pv. *oryzae* KACC10331	67.164
TC2-1_004461	XopT	*Xanthomonas fragariae* NBC2815	81.667
TC2-1_004471	XopT	*Xanthomonas fragariae* NBC2815	85
TC2-1_004490	XopT	*Xanthomonas fragariae* NBC2815	80
TC2-1_004495	XopT	*Xanthomonas fragariae* NBC2815	85
TC2-1_004498	AvrXccC	*Xanthomonas campestris* pv. *campestris* 8004	92.145
TC2-1_004521	XOO4824	*Xanthomonas oryzae* pv. *oryzae* KACC10331	70.769
TC2-1_004526	HopAO1	*Pseudomonas syringae* pv. *tomato* str. DC3000	32.845
TC2-1_004532	HopF4	*Pseudomonas savastanoi* NCPPB 3335	77.193
TC2-1_004536	AvrBs3	*Xanthomonas campestris* pv. *vesicatoria* 85-10	92.354
TC2-1_004538	XOO4824	*Xanthomonas oryzae* pv. *oryzae* KACC10331	67.164
TC2-1_004547	XOO4824	*Xanthomonas oryzae* pv. *oryzae* KACC10331	70.149
TC2-1_004562	XOO4824	*Xanthomonas oryzae* pv. *oryzae* KACC10331	64.179
TC2-1_004564	XOO4824	*Xanthomonas oryzae* pv. *oryzae* KACC10331	65.672
TC2-1_004566	XOO4824	*Xanthomonas oryzae* pv. *oryzae* KACC10331	65.672
TC2-1_004580	XOO4824	*Xanthomonas oryzae* pv. *oryzae* KACC10331	74.346
TC2-1_004589	XOO4824	*Xanthomonas oryzae* pv. *oryzae* KACC10331	68.254

## Discussion

The rapid advancements of high-throughput sequencing technologies and metagenomics in recent years have greatly promoted many types of biological studies, including plant pathology. In this study, we employed 16S rDNA gene-based metagenomic sequencing to understand the composition of microbiota associated with bacterial leaf canker of water spinach, which provides an important guide for pathogen identification. Metagenomic analysis showed that *Xanthomonas* is overwhelmingly dominant in all the diseased leaf samples, and *Pantoea* is also present in two of the leaf samples, which is in sharp contrast with rhizospheric soil and irrigation water samples (Figure 2). Species identification unveiled them as *X. perforans* and *P. ananatis* through complete genome sequencing and MLSA analysis, respectively.

Results from the metagenomic analysis revealed very low abundance of *Xanthomonas* (0.06 and 0.39%) and *Pantoea* (0∼0.15%) in water and soil samples. We cannot determine whether the *Xanthomonas* and *Pantoea* in water and soil can contribute to the disease since no isolation work has been performed from soil and water. However, from our observation on field planting, farmers prefer to reserve stumps from the previous year and plant them directly in the field; these old stumps could more likely be the primary infection source of the disease, and the pathogens can spread in large numbers in the field with wind and rain.

Inoculation of the bacteria revealed that both *X. perforans* and *P. ananatis* are pathogenic, causing distinctive diseased symptomatic features on water spinach leaves. Previously, *X. campestris* and *X. perforans* have been isolated from water spinach in Bang Pai, Thailand (Leksomboon et al., 1991), and Fuzhou, China (information from NCBI GenBank sequence records MN626337.1 to MN626340.1), respectively, causing bacterial leaf spot. Due to the lack of description of disease symptoms, we could not judge whether the disease symptoms caused by these two pathogens are consistent with the bacterial leaf canker caused by *X. perforans* strains reported in this study. Nevertheless, *X. perforans* has been reported to infect *Eucalyptus pellita* causing bacterial leaf blight and dieback (Bophela et al., 2019), with similar necrosis symptoms to the bacterial leaf canker of water spinach. Interestingly, this disease has also been reported to be caused by *P. ananatis* previously (Coutinho et al., 2002). Inoculation of either *X. perforans* or *P. ananatis* resulted in similar diseased symptoms on *Eucalyptus* leaves (Bophela et al., 2019), suggesting that both of the pathogens can infect the same host plant, although they have not been isolated from the same host sample simultaneously before this study.

In this study, both *X. perforans* and *P. ananatis* were isolated from the same sample of water spinach. Notably, there have been an increasing number of reports of co-infection in plant diseases. For instance, *P. ananatis* was found to synergistically infect *Eucalyptus* along with a fungal pathogen, *Corniothyrium zuluense*, causing serious canker disease (Van Zyl, 1999). Recently, we also isolated two bacterial pathogens from the same diseased rice sample, namely, *Enterobacter asburiae* and *P. ananatis*, both of which were determined as the causal agents of rice bacterial blight with no detectable synergistic or antagonistic interactions (Xue et al., 2020). Moreover, *Dickeya zeae* and *Stenotrophomonas maltophilia* have been isolated from the same tissue of soft rot leaf of *Clivia miniata* with no synergism (Hu et al., 2018, 2021). These, and many other cases, all suggest the common phenomenon of co-infection of two or more pathogens on host plants in nature.

The interaction between co-infected pathogens is synergistic, mutualistic, or antagonistic. Synergistic interactions of plant pathogens usually result in increased disease severity, the most typical example of which is the tomato pith necrosis caused by pathogen complexes including *Pseudomonas corrugata* (Scarlett et al., 1978), *Ps. mediterranea* (Catara et al., 2002), *Ps. marginalis* (Bella and Catara, 2009), *Ps. fluorescens*, *Ps. putida*, *Ps. citronellolis*, *Ps. straminea*, *Pantoea agglomerans*, and *X. perforans* (Aiello et al., 2013). The disease is greatly aggravated when co-inoculation with *Ps. corrugata* and *Ps. mediterranea* (Moura et al., 2005). In some cases, the synergism among pathogens could be indirect as for instance, some pathogens can suppress host immunity and promote colonization by other ones. On the other hand, antagonistic interaction between mixed pathogens often results from nutrition competition. In a study, growth interference was observed between different strains of *Ps. syringae* in a mixed infection (Bartoli et al., 2015). In our case, *X. perforans* is most likely the major pathogen based on its dramatically higher abundance and isolation rate, as well as its typical characteristics of crater-shaped ulcerative spots on leaves, whereas, *P. ananatis* is possibly a companion pathogen causing yellowing and brown rot lesions on leaves. In this study, we performed co-inoculation of *X. perforans* and *P. ananatis* on the host plant, and results showed that co-inoculation of both pathogens could aggravate the disease development under the condition of non-wound inoculation (Figure 4), suggesting that there may be synergistic interaction between *P. ananatis* TC3-1 and *X. perforans* TC2-1 under natural infection. From our observation, the main disease symptom in the field is the leaf canker caused by *Xanthomonas*, whereas *Pantoea* is also present in the field according to the results of 16S rDNA amplicon sequencing and laboratory microbial isolation toward symptomatic leaves, suggesting that *P. anantis* might behave as a latent pathogen within a susceptible leaf tissue. To further verify the pathogenicity of *P. ananatis* TC3-1, we inoculated it into tobacco leaves, and results showed that *P. ananatis* TC3-1 is able to induce hypersensitivity reaction (HR), although the HR response was not as strong as *X. perforans* TC2-1 (Supplementary Figure 5).

The genus *Xanthomonas* currently comprises over 35 species, most of which cause plant diseases in more than 400 different hosts (Timilsina et al., 2020). In 1991, *X. perforans* was first identified in tomato fields (Jones, 1995) and has outcompeted *X. euvesicatoria* by 2006 in Florida (Horvath et al., 2012). Until a single-strain Xp2010 was isolated from an infected pepper sample in 2010 in Florida (Schwartz et al., 2015), the host range of *X. perforans* was believed to be restricted to tomato. Findings in this study expand the host range of both *X. perforans* and *P. ananatis*. We also tested the pathogenicity of *X. perforans* TC2-1 on pepper, tomato, citrus leaves, and mango fruit, and found that strain TC2-1 could infect all of the above plants (Supplementary Figure 6). To identify genes that might be responsible for the host specificity of *X. perforans* TC2-1, its genome was sequenced and compared with the genomes of other *X. perforans*, which were all isolated from tomato and pepper. In total, 277 TC2-1-specific proteins were found, most of which encode transposases, suggesting a frequent genomic recombination of the strain, and more informatively, 23 predicted T3SEs were found to be absent in the other *X. perforans*-released genomes (Supplementary Table 8). The repertoire of T3SEs has been believed to be correlated with the host specificity of pathogenic *Xanthomonas* strains (Hajri et al., 2009, 2012a,b; Jacques et al., 2016). Whether these TC2-1 unique T3SEs play an important role in host specialization awaits further functional investigation.

## Data Availability Statement

The datasets presented in this study can be found in online repositories. The names of the repository/repositories and accession number(s) can be found in the article/Supplementary Material.

## Author Contributions

JZ conceived and designed the experiments. MH isolated and identified the pathogens and performed the pathogenicity tests. MH, YX, SW, AH, SC, and XM investigated the disease incidence. MH, CL, and XZ analyzed the metagenomic and genome data. MH, CL, JZ, and XZ wrote and revised the manuscript. All authors contributed to the article and approved the submitted version.

## Conflict of Interest

The authors declare that the research was conducted in the absence of any commercial or financial relationships that could be construed as a potential conflict of interest.

## Publisher’s Note

All claims expressed in this article are solely those of the authors and do not necessarily represent those of their affiliated organizations, or those of the publisher, the editors and the reviewers. Any product that may be evaluated in this article, or claim that may be made by its manufacturer, is not guaranteed or endorsed by the publisher.

## Footnotes

1
https://github.com/marcelm/cutadapt/

2
http://www.drive5.com/usearch/

3
https://github.com/OpenGene/fastp

4
http://www.r-project.org/
