# Supplementary material for: Microbial Diversity Analysis and Genome Sequencing Identify Xanthomonas perforans as the Pathogen of Bacterial Leaf Canker of Water Spinach (Ipomoea aquatic)
Source: Front Microbiol. 2021 Oct 27;12:752760. doi: 10.3389/fmicb.2021.752760 (PMC8579042; doi:10.3389/fmicb.2021.752760)
Supplement: Supplementary file 3 [file Data_Sheet_1.PDF]

Supplemental Material

Microbial diversity analysis and genome sequencing identify *Xanthomonas perforans* as the pathogen of bacterial leaf canker of water spinach (*Ipomoea aquatic*)

Ming Hu[1,#], Chuhao Li[1,#], Xiaofan Zhou[1,#], Yang Xue[1], Si Wang[1], Anqun Hu[1], Shanshan Chen[1], Xiuwen Mo[2], Jianuan Zhou[1],*

[1] Guangdong Laboratory for Lingnan Modern Agriculture, Guangdong Province Key Laboratory of Microbial Signals and Disease Control, Integrative Microbiology Research Centre, South China Agricultural University, Guangzhou 510642, China

[2] Agricultural Technology Service Centre of Daojiao Town, Dongguan 523170, China

***** Corresponding author: jianuanzhou@scau.edu.cn

Contents:

Supplementary Table S1.

Supplementary Table S2.

Supplementary Table S3.

Supplementary Table S4.

Supplementary Table S5.

Supplementary Table S6.

Supplementary Fig. S1

Supplementary Fig. S2

Supplementary Fig. S3

Supplementary Fig. S4

Supplementary Fig. S5

Supplementary Fig. S6

Table S1. Primers used in this study

Gene	Primer	Primer sequence (5'-3')	Reference
16S rDNA	27f	AGAGTTTGATCCTGGCTCAG	Coenye et al., 1999
	1492r	TACGGCTACCTTGTTACGACTT	
atpD	atpD 01-F	RTAATYGGMGCSGTRGTNGAYGT	Brady et al., 2008
	atpD 08-R	TCATCCGCMGGWACRTAWAYNGCCTG	
avrBs2	XAvrBs2-F	GGACTAGTCCTGCCGGTGTTGATGCACGA	Hajri et al., 2009
	XAvrBs2-R	CCGCTCGAGCGGTGATCGGTCAACAGGCTTTC	
cpn60	H1594F	GACGTCGCCGGTGACGGCACCACCAC	Hill et al., 2004; Tian, 2018
	H1595R	CGACGGTCGCCGAAGCCCGGGGCCTT	
gyrB	gyrB 01-F	TAARTTYGAYGAYAACTCYTAYAAAGT	Brady et al., 2008
	gyrB 02-R	CMCCYTCCACCARGTAMAGTT	
	XgyrB-f	AAGCAGGGCAAGAGCGAGCTGTA	Parkinson et al., 2007
	XgyrB-r	CAAGGTGCTGAAGATCTGGTC	
infB	infB 05-F	ATYATGGGHCAYGTHGAYCA	Brady et al., 2008
	infB 02-R	ACKGAGTARTAACGCAGATCCA	
rpoB	rpoB CM7-F	AACCAGTTCCGCGTTGGCCTG	Brady et al., 2008
	rpoB CM31b-R	CCTGAACAACACGCTCGGA	
rpoD	XrpoD1F	TGGAACAGGGCTATCTGACC	Tian, 2018
	XrpoD1R	CATTCYAGGTTGGTCTGRTT	

Table S2. Accession nos. of the gene sequences of the obtained isolates

Isolate No.	16S rDNA	*atpD*	*avrBs2*	*cpn60*	*gyrB*	*infB*	*rpoB*	*rpoD*
TC1-1	MT998932	MW436583	MW436587	MW436589	MW436591	/	/	MW436599
TC2-1	MT998933	MW436584	MW436588	MW436590	MW436592	/	/	MW436600
TC3-1	MT998934	MW436585	/	/	MW436593	MW436595	MW436597	/
TC3-2	MT998935	MW436586	/	/	MW436594	MW436596	MW436598	/

Table S3. Incidence of bacterial leaf spot of water spinach in Dongguan City (2019-2020)

Cultivar	Time	Place	Area (m^2)	Incidence (%)[a]
Baijun 311	11 Jul. 2019	Changping Village, Daojiao	200	100
Baijun 311	11 Jul. 2019	Dayusha Village, Daojiao	133	100
Baijun 611	5 Aug. 2019	Jiaolian Zone, Wanjiang	400	100
Baijun 611	28 Jun. 2020	Dafen Zone, Wanjiang	667	100
Baijun 311	18 Sep. 2020	Baoanwei Village, Gaobu	2667	100
Baijun 311	21 Sep. 2020	Caibaibailu Village, Daojiao	333	100
Qingtong 322	21 Sep. 2020	Caibaibailu Village, Daojiao	67	16.7
Baijun 311	27 Sep. 2020	Dafen Zone, Wanjiang	667	100
Qingtong 322	27 Sep. 2020	Dafen Zone, Wanjiang	67	100
Qingtong 322	28 Sep. 2020	Baoanwei Village, Gaobu	2000	86

[a]Incidence was calculated as the percentage of infected clumps in total clumps surveyed.

Table S4. Reads obtained from 16S rDNA amplicon sequencing

Sample ID	High-quality	Low-quality	Host-contamination	Total	High-quality percentage
DF1	18546	7505	57949	84000	22.08%
DF2	2716	7169	74115	84000	3.23%
DF3	14548	7722	61730	84000	17.32%
DF4	21612	7948	54404	83964	25.74%
DFw	70968	7736	4952	83656	84.83%
DFs1	46539	36658	803	84000	55.40%
DFs2	41479	41193	1328	84000	49.38%
DFs3	45874	36832	1294	84000	54.61%

Table S5. The relative abundances of the top 30 OTUs at genus level

Genus	DF1	DF2	DF3	DF4	DFw	DFs1	DFs2	DFs3
(Unassigned)	0.003295338	0.017222014	0.005871788	0.005974435	0.217259428	0.293693189	0.408291914	0.408583841
Xanthomonas	0.98881746	0.951703482	0.886363636	0.97086884	7.05E-05	6.46E-05	0.000387926	0.000327461
uncultured	0.000540219	0.002620741	0.000621719	0.000370508	0.109123485	0.23756085	0.183028246	0.158229092
hgcI_clade	0.000162066	0	6.91E-05	0	0.156186956	0.000258476	0.000218208	0.000240138
Polynucleobacter	0	0	0	0	0.080795113	0.001055443	0.000751606	0.001069706
Chujaibacter	0	0	0	0	2.82E-05	0.072071684	0.007782762	0.044752985
*Cyanobium*_PCC-6307	0	0	0	0	0.073078171	0.000258476	0.00036368	0.000261969
Sediminibacterium	0	0	0	0.00013894	0.038937404	0.007517339	0.003418596	0.002663348
Pseudarcicella	0	0	0	0	0.046555592	4.31E-05	0	4.37E-05
Rhodanobacter	0.000108044	0	0.000276319	0.00013894	2.82E-05	0.029358549	0.010740696	0.028423603
C39	0	0	0	0	0.034733293	6.46E-05	0	6.55E-05
Sphingomonas	0.000108044	0	0.000276319	0.00013894	0.000239832	0.01621936	0.022814887	0.015914598
CL500-29_marine_group	0	0	6.91E-05	0	0.032334975	6.46E-05	4.85E-05	6.55E-05
Bryobacter	0	0	0.00013816	4.63E-05	0.000155185	0.014130013	0.014571463	0.018621608
Nitrospira	0.000162066	0	0.000345399	0.00013894	0.000451448	0.005643389	0.03236756	0.009998472
Candidatus_Koribacter	0	0	6.91E-05	4.63E-05	2.82E-05	0.010920605	0.017092981	0.016918812
*Alkalinema*_CENA528	0	0	0	0	0.000141078	0.019234911	0.008243423	0.013906172
FCPS473	0	0	0	0	0	0.014431569	0.004824827	0.021219464
Fluviicola	0	0	0	0	0.021824697	0.001378538	0.000242454	4.37E-05
Dinghuibacter	0	0	0	0	0.020413922	0.000215397	0.000921324	0.000109154
Candidatus_Solibacter	0	0	0.00013816	4.63E-05	8.46E-05	0.005643389	0.014862408	0.012814635
Gemmatimonas	0	0	0	4.63E-05	0.001650607	0.006031103	0.016171657	0.007684415
Pseudomonas	0.000216088	0.000374392	0.006700746	0.000370508	0.001029866	0.01682247	0.003806522	0.005894296

Candidatus_Udaeobacter	0	0	0	0	0	0.011523715	0.014474482	0.005195712
Pantoea	0	0	0.06265543	0.011578362	0	0.001464697	2.42E-05	0.000174646
Terrimonas	0	0	0	4.63E-05	0.011610682	0.000689269	0.003515578	0.002183072
Haliangium	0	0	0.00013816	0.00013894	0.000112862	0.00439409	0.00867984	0.008557644
Flavobacterium	0	0	0	0	0.006433136	0.004480248	0.002473027	0.003776715
Acidibacter	0	0	0	4.63E-05	0.011201557	0.001120062	0.000606134	0.001397166
Castellaniella	0	0	0	0	0	0.008874338	0.000606134	0.009474534
others	0.006490676	0.027979371	0.036166925	0.009764765	0.135390879	0.214671895	0.21856893	0.201288434

Table S6. Bacterial isolates and tentative taxonomic status according to 16S rDNA sequences

Isolate No.	Source	Tissue	Tentative taxonomic status
TC1-1	Field of Jiaxing Vegetable Planting Cooperative, Dafen Zone, Wanjiang	Leaf 1	*Xanthomonas* sp.
TC1-2			*Xanthomonas* sp.
TC1-3			*Pseudomonas* sp.
TC1-4			*Bacillus aquimaris*
TCX11			*Ps. aeruginosa*
TC2-1		Leaf 2	*Xanthomonas* sp.
TC2-2			*Bacillus* sp.
TC2-3			*Xanthomonas* sp.
TC2-4			*Bacillus* sp.
TCX21			*Xanthomonas* sp.
TCX22			*Bacillus* sp.
TC3-1		Leaf 3	*Pantoea* sp.
TC3-2			*Pantoea* sp.
TC3-3			*Pseudomonas* sp.
TC3-4			*Pseudomonas* sp.
TC3-5			*Pantoea* sp.
TCX31			*Bacillus* sp.
TCX32			*Bacillus* sp.
TCX33			*Bacillus* sp.
WJ1		Leaf 4	*Bacillus* sp.
WJ2			*Bacillus* sp.
WJ2-1		Leaf 5	*Bacillus* sp.
WJ2-2			*Bacillus* sp.

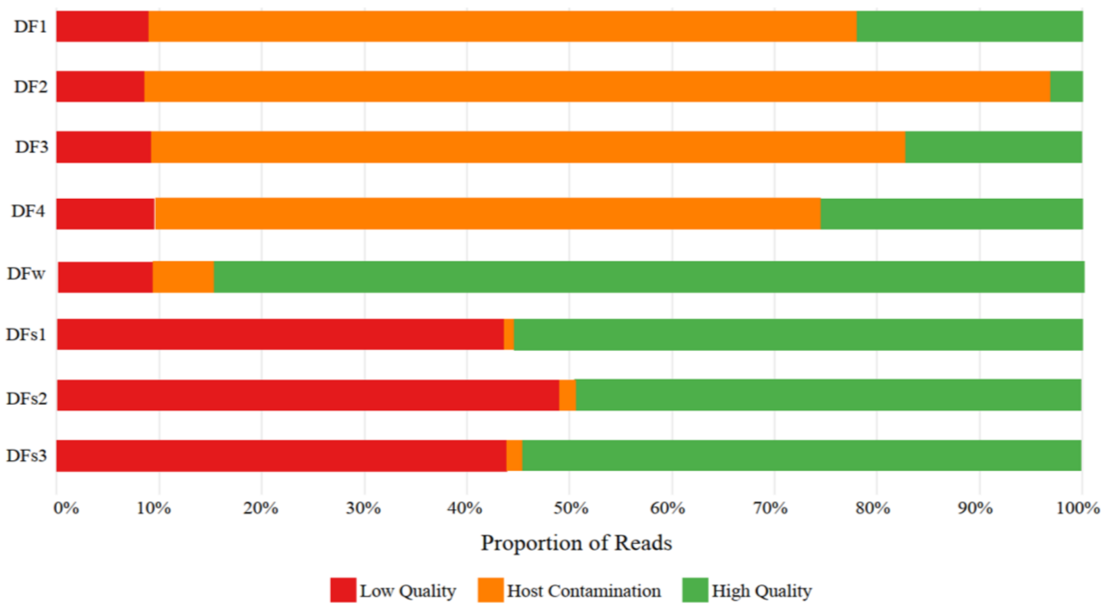

Fig. S1. Outputs of the metagenomic reads obtained in this study. Stacked bar-chart indicating the proportion of reads from each sample discarded due to low quality (red), host genome (orange), and quality filtered reads (green) obtained for downstream analysis.

A

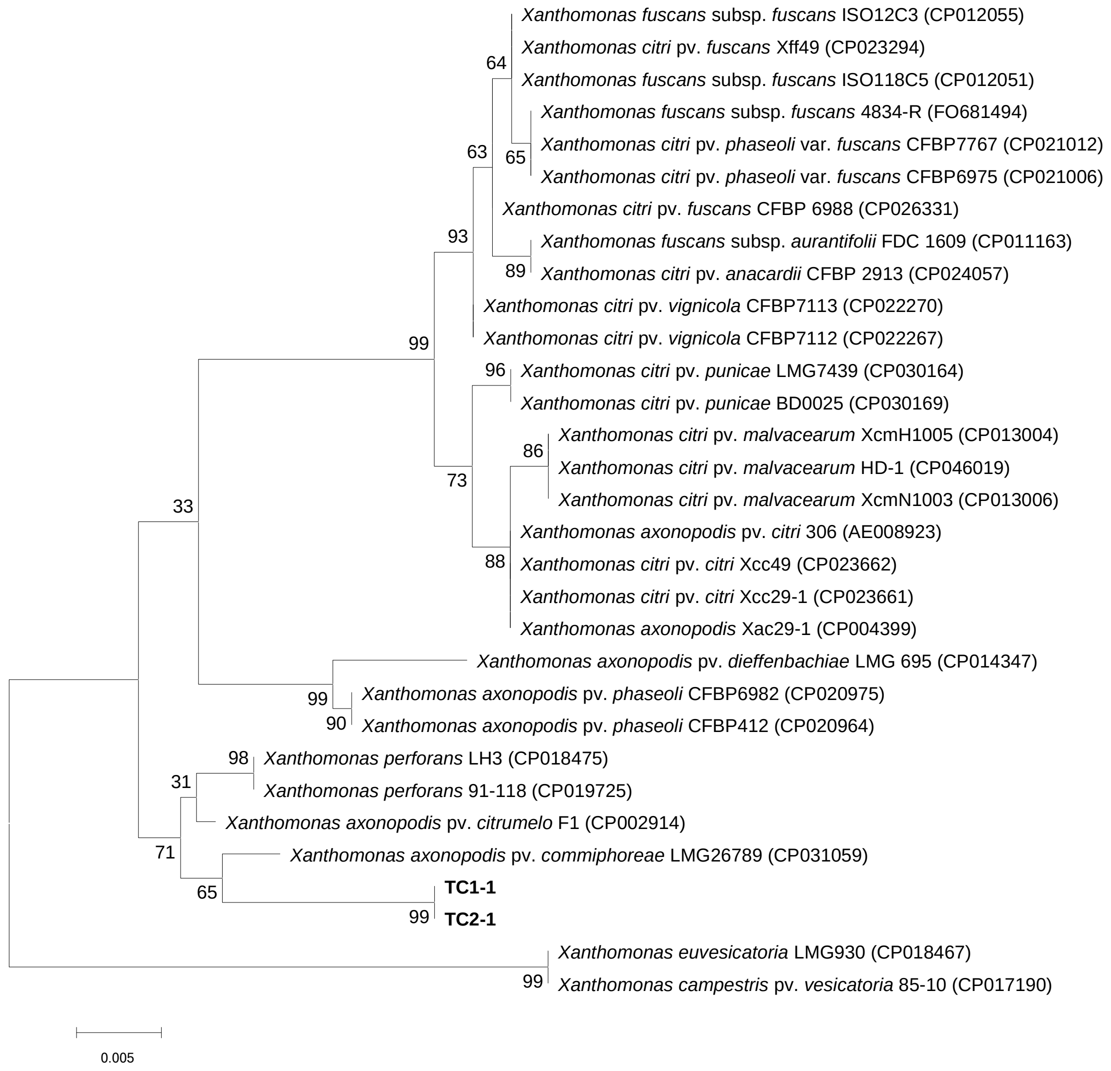
Xanthomonas fuscans subsp. fuscans ISO12C3 (CP012055)
Xanthomonas citri pv. fuscans Xff49 (CP023294)
Xanthomonas fuscans subsp. fuscans ISO118C5 (CP012051)
Xanthomonas fuscans subsp. fuscans 4834-R (FO681494)
Xanthomonas citri pv. phaseoli var. fuscans CFBP7767 (CP021012)
Xanthomonas citri pv. phaseoli var. fuscans CFBP6975 (CP021006)
Xanthomonas citri pv. fuscans CFBP 6988 (CP026331)
Xanthomonas fuscans subsp. aurantifolii FDC 1609 (CP011163)
Xanthomonas citri pv. anacardii CFBP 2913 (CP024057)
Xanthomonas citri pv. vignicola CFBP7113 (CP022270)
Xanthomonas citri pv. vignicola CFBP7112 (CP022267)
Xanthomonas citri pv. punicae LMG7439 (CP030164)
Xanthomonas citri pv. punicae BD0025 (CP030169)
Xanthomonas citri pv. malvacearum XcmH1005 (CP013004)
Xanthomonas citri pv. malvacearum HD-1 (CP046019)
Xanthomonas citri pv. malvacearum XcmN1003 (CP013006)
Xanthomonas axonopodis pv. citri 306 (AE008923)
Xanthomonas citri pv. citri Xcc49 (CP023662)
Xanthomonas citri pv. citri Xcc29-1 (CP023661)
Xanthomonas axonopodis Xac29-1 (CP004399)
Xanthomonas axonopodis pv. dieffenbachiae LMG 695 (CP014347)
Xanthomonas axonopodis pv. phaseoli CFBP6982 (CP020975)
Xanthomonas axonopodis pv. phaseoli CFBP412 (CP020964)
Xanthomonas perforans LH3 (CP018475)
Xanthomonas perforans 91-118 (CP019725)
Xanthomonas axonopodis pv. citrumelo F1 (CP002914)
Xanthomonas axonopodis pv. commiphoreae LMG26789 (CP031059)
TC1-1
TC2-1
Xanthomonas euvesicatoria LMG930 (CP018467)
Xanthomonas campestris pv. vesicatoria 85-10 (CP017190)
64
65
63
93
89
99
96
86
73
88
33
99
90
98
31
71
65
99
99
0.005

B

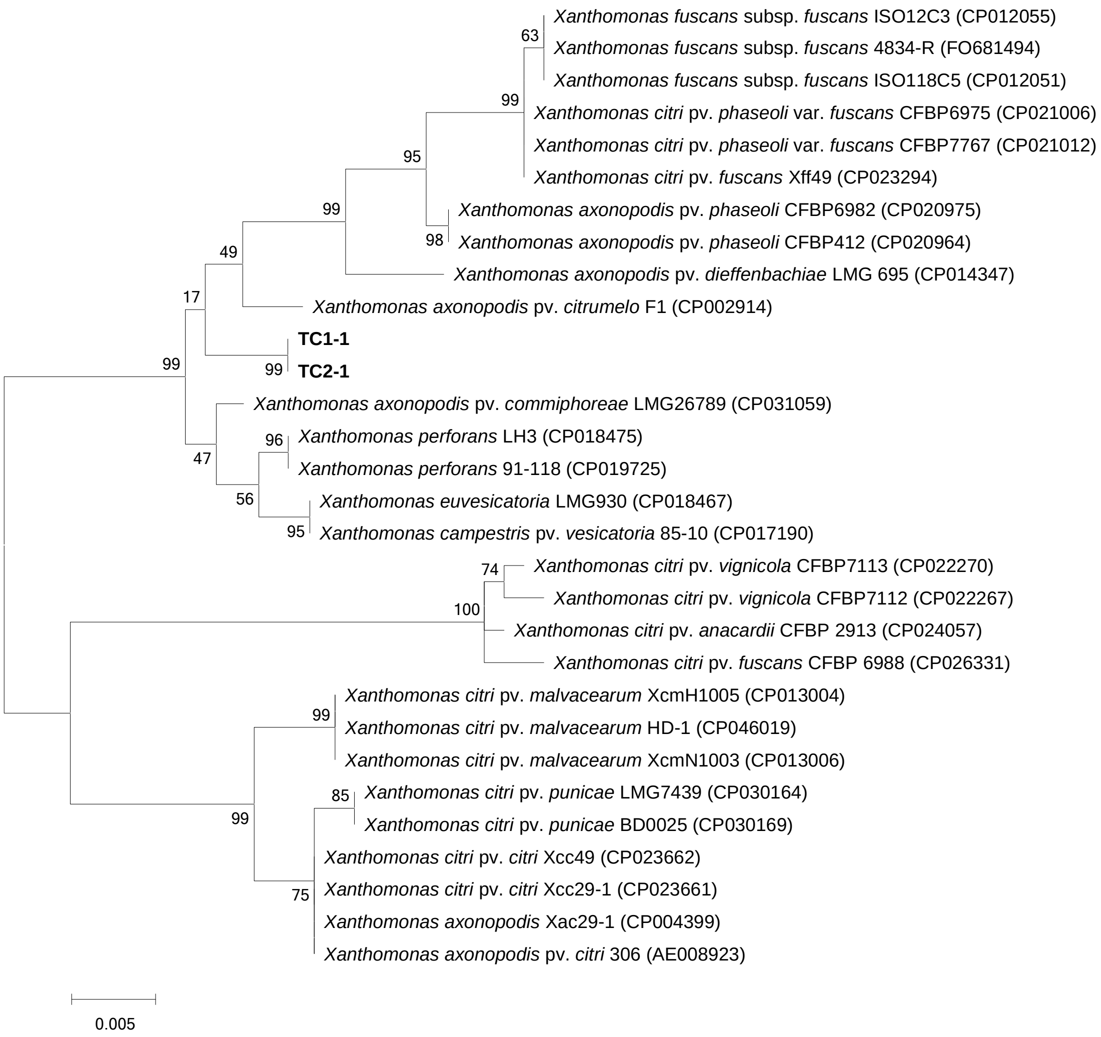
Xanthomonas fuscans subsp. fuscans ISO12C3 (CP012055)
Xanthomonas fuscans subsp. fuscans 4834-R (FO681494)
Xanthomonas fuscans subsp. fuscans ISO118C5 (CP012051)
Xanthomonas citri pv. phaseoli var. fuscans CFBP6975 (CP021006)
Xanthomonas citri pv. phaseoli var. fuscans CFBP7767 (CP021012)
Xanthomonas citri pv. fuscans Xff49 (CP023294)
Xanthomonas axonopodis pv. phaseoli CFBP6982 (CP020975)
Xanthomonas axonopodis pv. phaseoli CFBP412 (CP020964)
Xanthomonas axonopodis pv. dieffenbachiae LMG 695 (CP014347)
Xanthomonas axonopodis pv. citrumelo F1 (CP002914)
TC1-1
TC2-1
Xanthomonas axonopodis pv. commiphoreae LMG26789 (CP031059)
Xanthomonas perforans LH3 (CP018475)
Xanthomonas perforans 91-118 (CP019725)
Xanthomonas euvesicatoria LMG930 (CP018467)
Xanthomonas campestris pv. vesicatoria 85-10 (CP017190)
Xanthomonas citri pv. vignicola CFBP7113 (CP022270)
Xanthomonas citri pv. vignicola CFBP7112 (CP022267)
Xanthomonas citri pv. anacardii CFBP 2913 (CP024057)
Xanthomonas citri pv. fuscans CFBP 6988 (CP026331)
Xanthomonas citri pv. malvacearum XcmH1005 (CP013004)
Xanthomonas citri pv. malvacearum HD-1 (CP046019)
Xanthomonas citri pv. malvacearum XcmN1003 (CP013006)
Xanthomonas citri pv. punicae LMG7439 (CP030164)
Xanthomonas citri pv. punicae BD0025 (CP030169)
Xanthomonas citri pv. citri Xcc49 (CP023662)
Xanthomonas citri pv. citri Xcc29-1 (CP023661)
Xanthomonas axonopodis Xac29-1 (CP004399)
Xanthomonas axonopodis pv. citri 306 (AE008923)
63
99
95
99
98
49
17
99
99
96
47
56
95
74
100
99
85
99
75
0.005

C

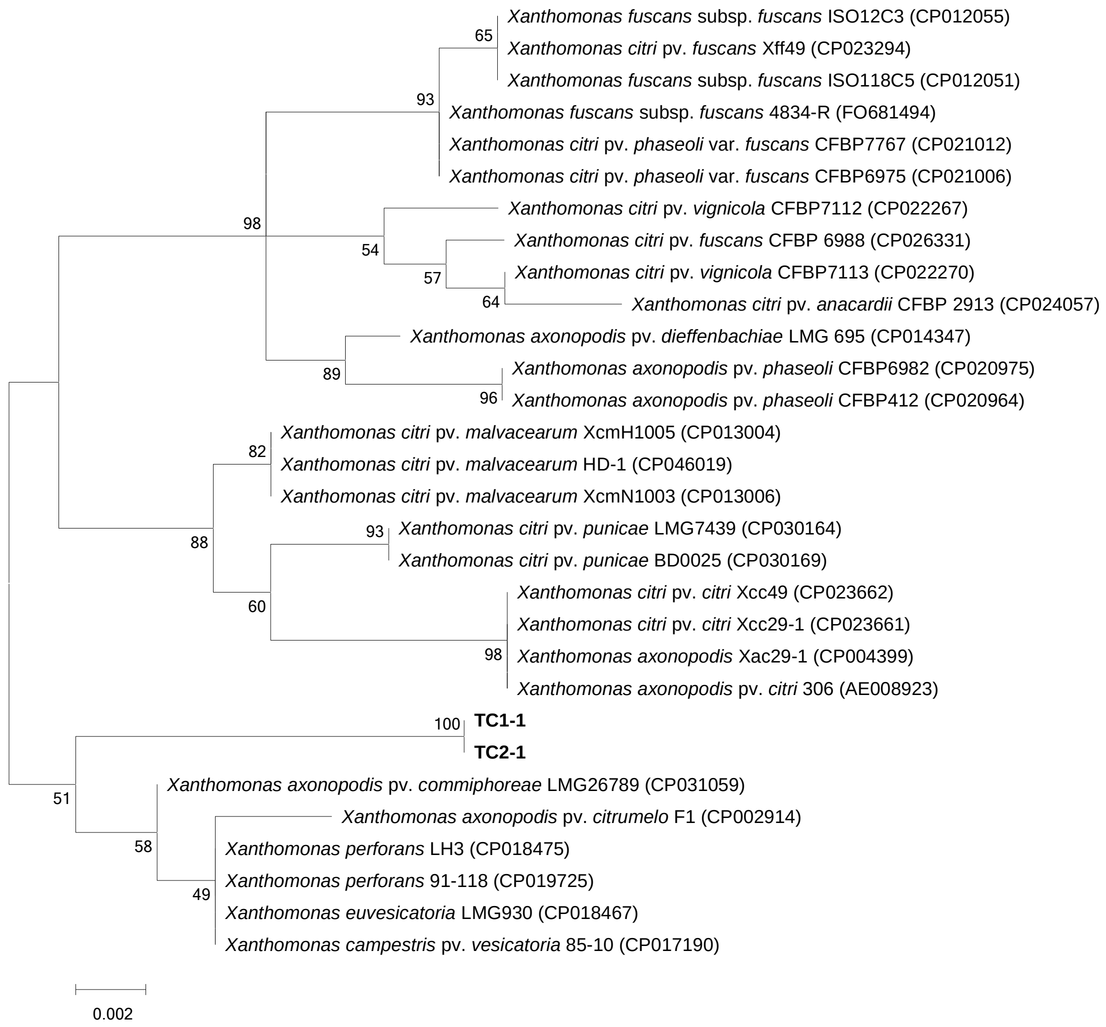

Xanthomonas fuscans subsp. fuscans ISO12C3 (CP012055)
Xanthomonas citri pv. fuscans Xff49 (CP023294)
Xanthomonas fuscans subsp. fuscans ISO118C5 (CP012051)
Xanthomonas fuscans subsp. fuscans 4834-R (FO681494)
Xanthomonas citri pv. phaseoli var. fuscans CFBP7767 (CP021012)
Xanthomonas citri pv. phaseoli var. fuscans CFBP6975 (CP021006)
Xanthomonas citri pv. vignicola CFBP7112 (CP022267)
Xanthomonas citri pv. fuscans CFBP 6988 (CP026331)
Xanthomonas citri pv. vignicola CFBP7113 (CP022270)
Xanthomonas citri pv. anacardii CFBP 2913 (CP024057)
Xanthomonas axonopodis pv. dieffenbachiae LMG 695 (CP014347)
Xanthomonas axonopodis pv. phaseoli CFBP6982 (CP020975)
Xanthomonas axonopodis pv. phaseoli CFBP412 (CP020964)
Xanthomonas citri pv. malvacearum XcmH1005 (CP013004)
Xanthomonas citri pv. malvacearum HD-1 (CP046019)
Xanthomonas citri pv. malvacearum XcmN1003 (CP013006)
Xanthomonas citri pv. punicae LMG7439 (CP030164)
Xanthomonas citri pv. punicae BD0025 (CP030169)
Xanthomonas citri pv. citri Xcc49 (CP023662)
Xanthomonas citri pv. citri Xcc29-1 (CP023661)
Xanthomonas axonopodis Xac29-1 (CP004399)
Xanthomonas axonopodis pv. citri 306 (AE008923)
TC1-1
TC2-1
Xanthomonas axonopodis pv. commiphoreae LMG26789 (CP031059)
Xanthomonas axonopodis pv. citrumelo F1 (CP002914)
Xanthomonas perforans LH3 (CP018475)
Xanthomonas perforans 91-118 (CP019725)
Xanthomonas euvesicatoria LMG930 (CP018467)
Xanthomonas campestris pv. vesicatoria 85-10 (CP017190)
65
93
98
54
57
64
89
96
82
88
93
60
98
100
51
58
49
0.002

D

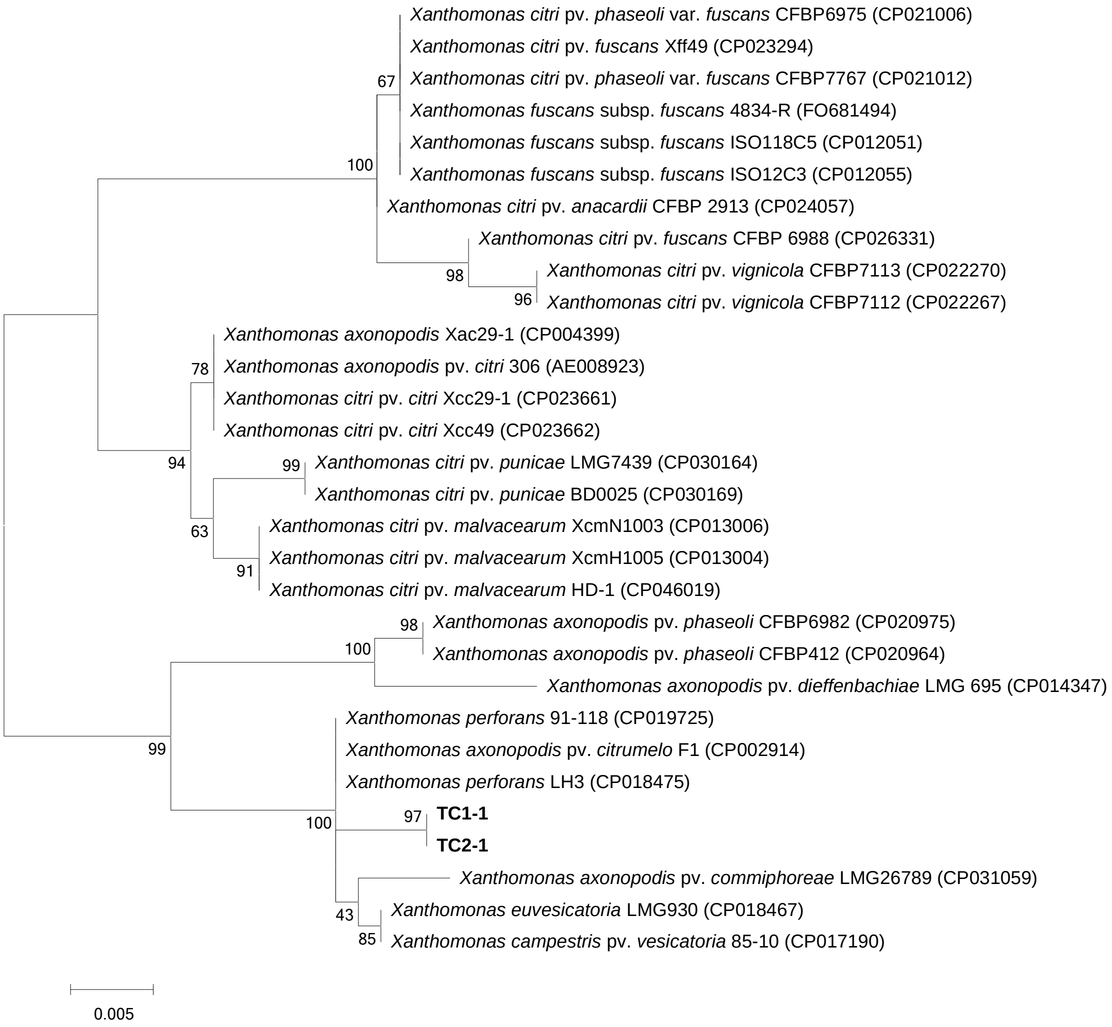

Xanthomonas citri pv. phaseoli var. fuscans CFBP6975 (CP021006)
Xanthomonas citri pv. fuscans Xff49 (CP023294)
Xanthomonas citri pv. phaseoli var. fuscans CFBP7767 (CP021012)
Xanthomonas fuscans subsp. fuscans 4834-R (FO681494)
Xanthomonas fuscans subsp. fuscans ISO118C5 (CP012051)
Xanthomonas fuscans subsp. fuscans ISO12C3 (CP012055)
Xanthomonas citri pv. anacardii CFBP 2913 (CP024057)
Xanthomonas citri pv. fuscans CFBP 6988 (CP026331)
Xanthomonas citri pv. vignicola CFBP7113 (CP022270)
Xanthomonas citri pv. vignicola CFBP7112 (CP022267)
Xanthomonas axonopodis Xac29-1 (CP004399)
Xanthomonas axonopodis pv. citri 306 (AE008923)
Xanthomonas citri pv. citri Xcc29-1 (CP023661)
Xanthomonas citri pv. citri Xcc49 (CP023662)
Xanthomonas citri pv. punicae LMG7439 (CP030164)
Xanthomonas citri pv. punicae BD0025 (CP030169)
Xanthomonas citri pv. malvacearum XcmN1003 (CP013006)
Xanthomonas citri pv. malvacearum XcmH1005 (CP013004)
Xanthomonas citri pv. malvacearum HD-1 (CP046019)
Xanthomonas axonopodis pv. phaseoli CFBP6982 (CP020975)
Xanthomonas axonopodis pv. phaseoli CFBP412 (CP020964)
Xanthomonas axonopodis pv. dieffenbachiae LMG 695 (CP014347)
Xanthomonas perforans 91-118 (CP019725)
Xanthomonas axonopodis pv. citrumelo F1 (CP002914)
Xanthomonas perforans LH3 (CP018475)
TC1-1
TC2-1
Xanthomonas axonopodis pv. commiphoreae LMG26789 (CP031059)
Xanthomonas euvesicatoria LMG930 (CP018467)
Xanthomonas campestris pv. vesicatoria 85-10 (CP017190)
67
100
98
96
78
94
99
63
91
98
100
99
100
97
43
85
0.005

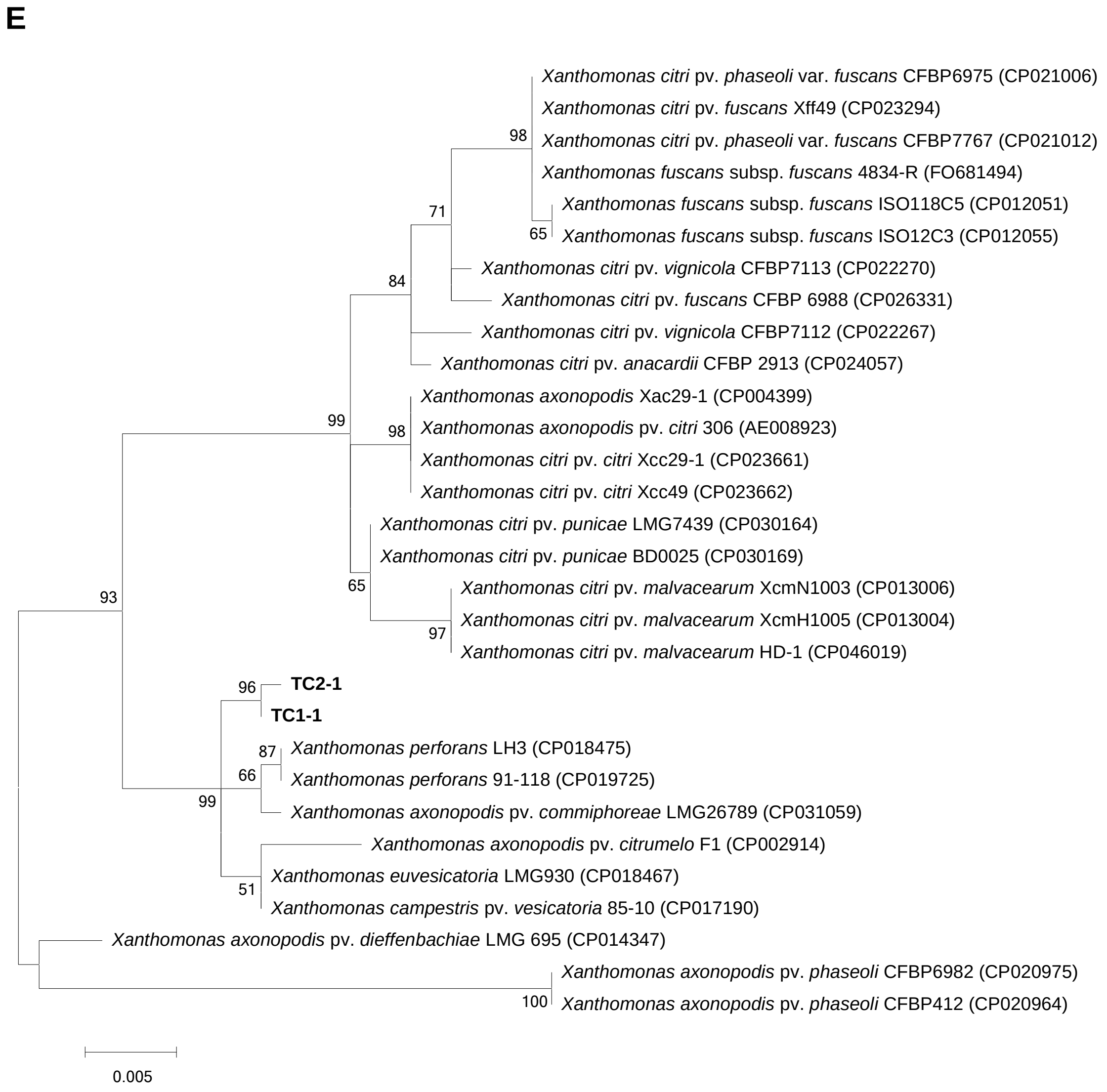
E
Xanthomonas citri pv. phaseoli var. fuscans CFBP6975 (CP021006)
Xanthomonas citri pv. fuscans Xff49 (CP023294)
Xanthomonas citri pv. phaseoli var. fuscans CFBP7767 (CP021012)
Xanthomonas fuscans subsp. fuscans 4834-R (FO681494)
Xanthomonas fuscans subsp. fuscans ISO118C5 (CP012051)
Xanthomonas fuscans subsp. fuscans ISO12C3 (CP012055)
Xanthomonas citri pv. vignicola CFBP7113 (CP022270)
Xanthomonas citri pv. fuscans CFBP 6988 (CP026331)
Xanthomonas citri pv. vignicola CFBP7112 (CP022267)
Xanthomonas citri pv. anacardii CFBP 2913 (CP024057)
Xanthomonas axonopodis Xac29-1 (CP004399)
Xanthomonas axonopodis pv. citri 306 (AE008923)
Xanthomonas citri pv. citri Xcc29-1 (CP023661)
Xanthomonas citri pv. citri Xcc49 (CP023662)
Xanthomonas citri pv. punicae LMG7439 (CP030164)
Xanthomonas citri pv. punicae BD0025 (CP030169)
Xanthomonas citri pv. malvacearum XcmN1003 (CP013006)
Xanthomonas citri pv. malvacearum XcmH1005 (CP013004)
Xanthomonas citri pv. malvacearum HD-1 (CP046019)
TC2-1
TC1-1
Xanthomonas perforans LH3 (CP018475)
Xanthomonas perforans 91-118 (CP019725)
Xanthomonas axonopodis pv. commiphoreae LMG26789 (CP031059)
Xanthomonas axonopodis pv. citrumelo F1 (CP002914)
Xanthomonas euvesicatoria LMG930 (CP018467)
Xanthomonas campestris pv. vesicatoria 85-10 (CP017190)
Xanthomonas axonopodis pv. dieffenbachiae LMG 695 (CP014347)
Xanthomonas axonopodis pv. phaseoli CFBP6982 (CP020975)
Xanthomonas axonopodis pv. phaseoli CFBP412 (CP020964)
98
65
71
84
99
98
65
97
93
96
87
66
99
51
100
0.005

F

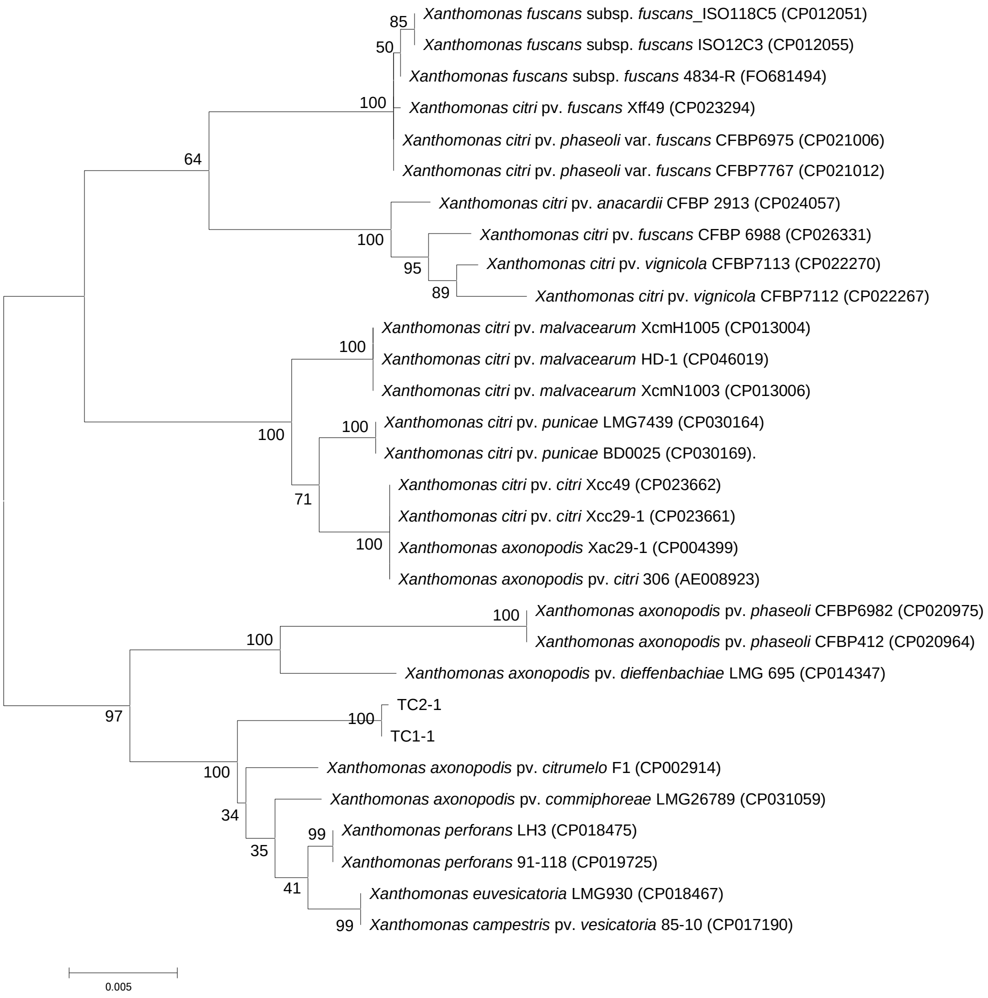

Fig. S2. Phylogenetic trees based on the concatenated nucleotide sequences of the *atpD* (A), *avrBs2* (B), *cpn60* (C), *gyrB* (D) and *rpoD* (E), and their joint sequences (F) for isolates TC1-1 and TC2-1 and their corresponding close related strains. Consensus sequences of each gene were aligned with ClustalW and trimmed in the same size and assembled to construct a Maximum-Likelihood tree. Bootstrap values after 1000 replicates are expressed as percentages.

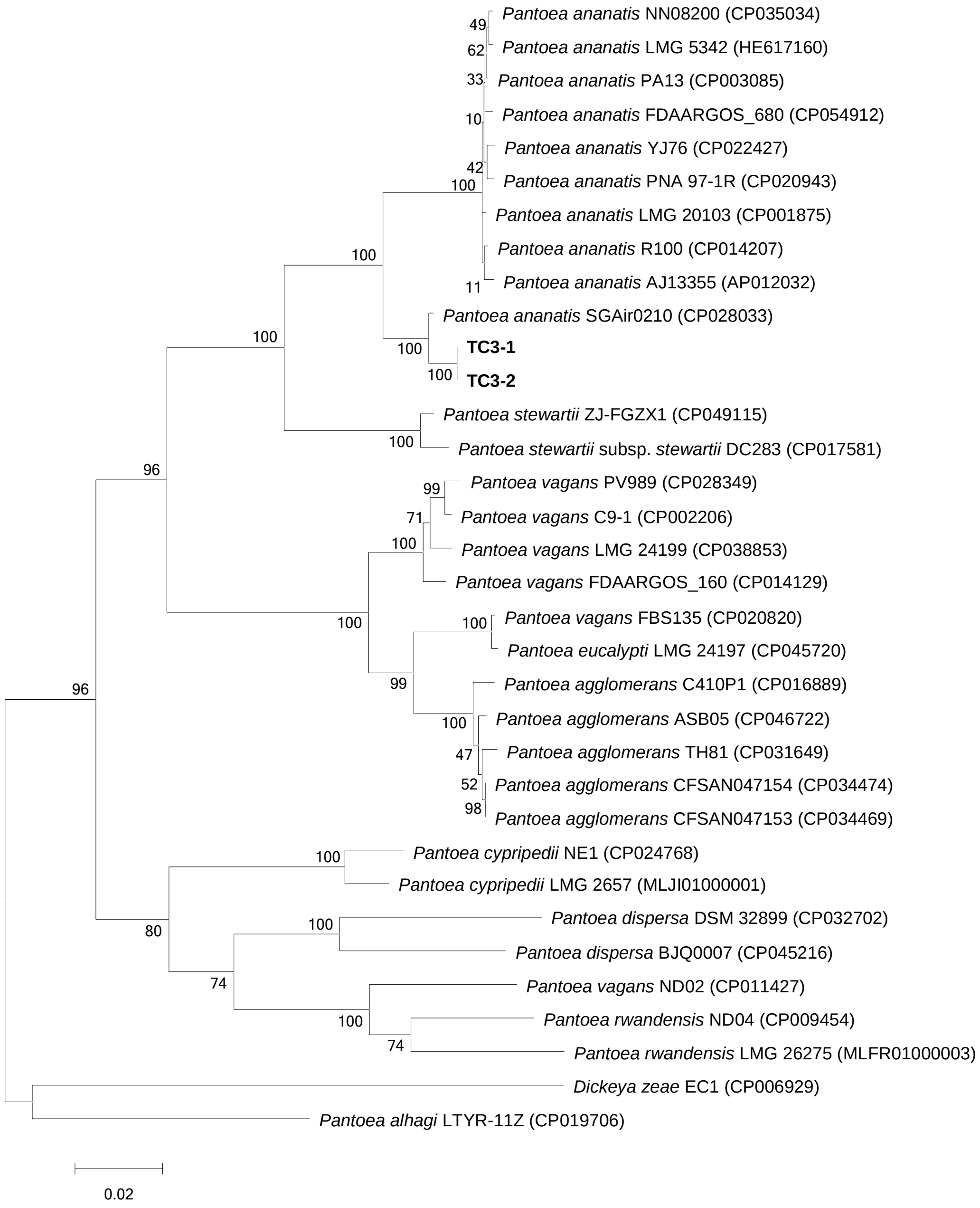

Fig. S3. Phylogenetic tree based on the joint concatenated nucleotide sequences of the *atpD*, *gyrB*, *infB* and *rpoB* genes for isolates TC3-1 and TC3-2 and their corresponding close related strains. Consensus sequences of each gene were aligned with ClustalW and trimmed in the same size and assembled to construct a Maximum-Likelihood tree. Bootstrap values after 1000 replicates are expressed as percentages.

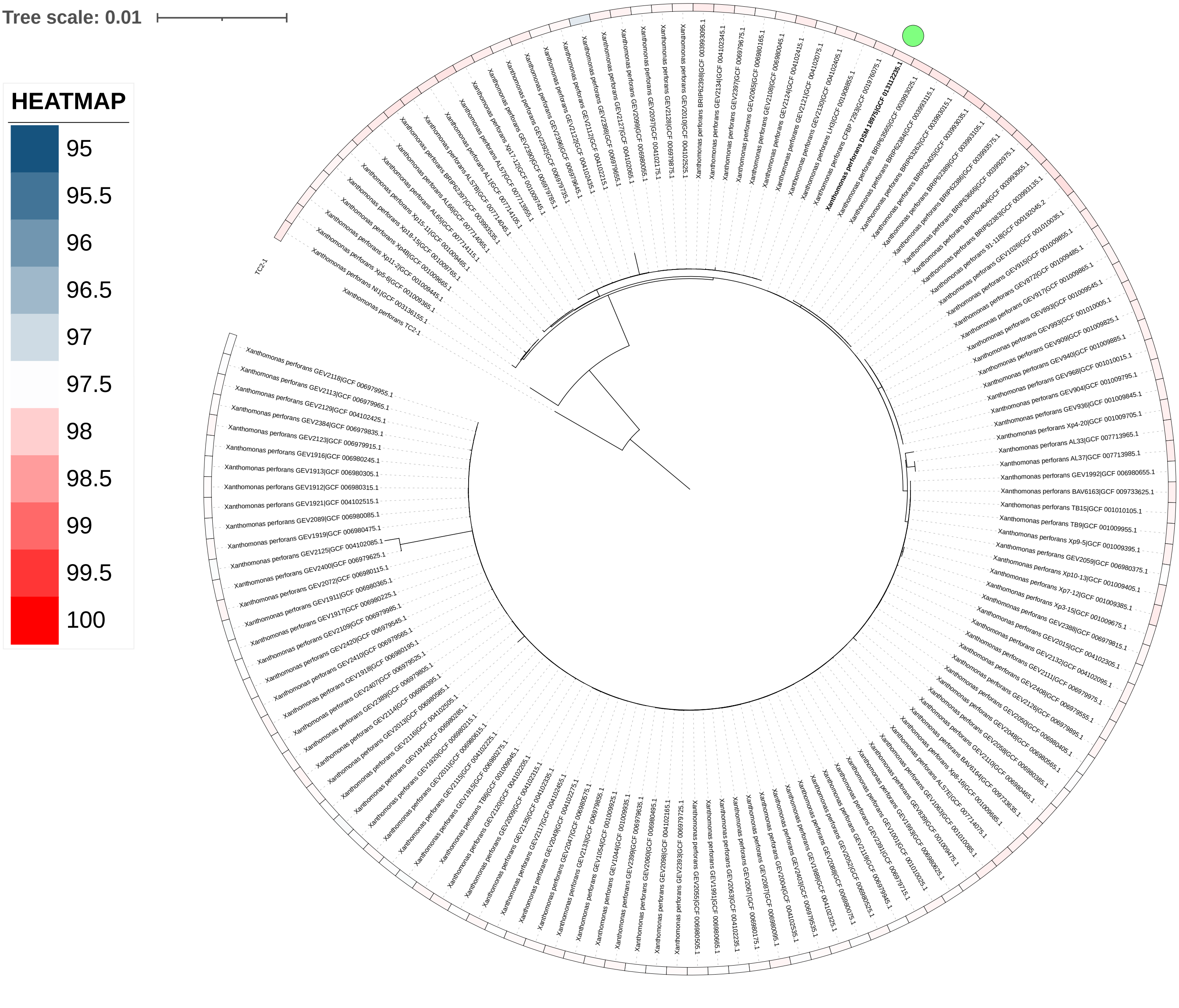

Fig. S4. Phylogenetic tree of TC2-1 and all the 141 sequenced *Xanthomonas perforans* strains in the NCBI RefSeq database based on 120 conserved, single-copy genes.

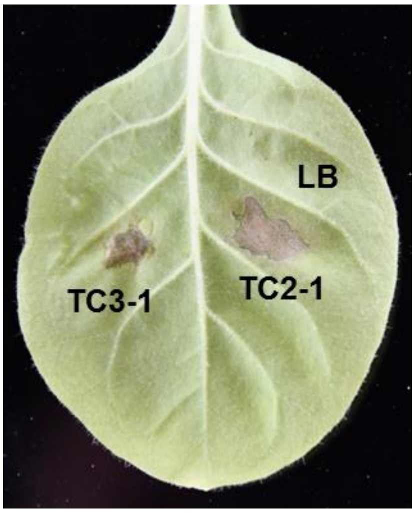

Fig. S5. Reaction of tobacco plant (*Nicotiana tabacum* variant K326) 2 days after infiltration with LB, TC2-1 and TC3-1.

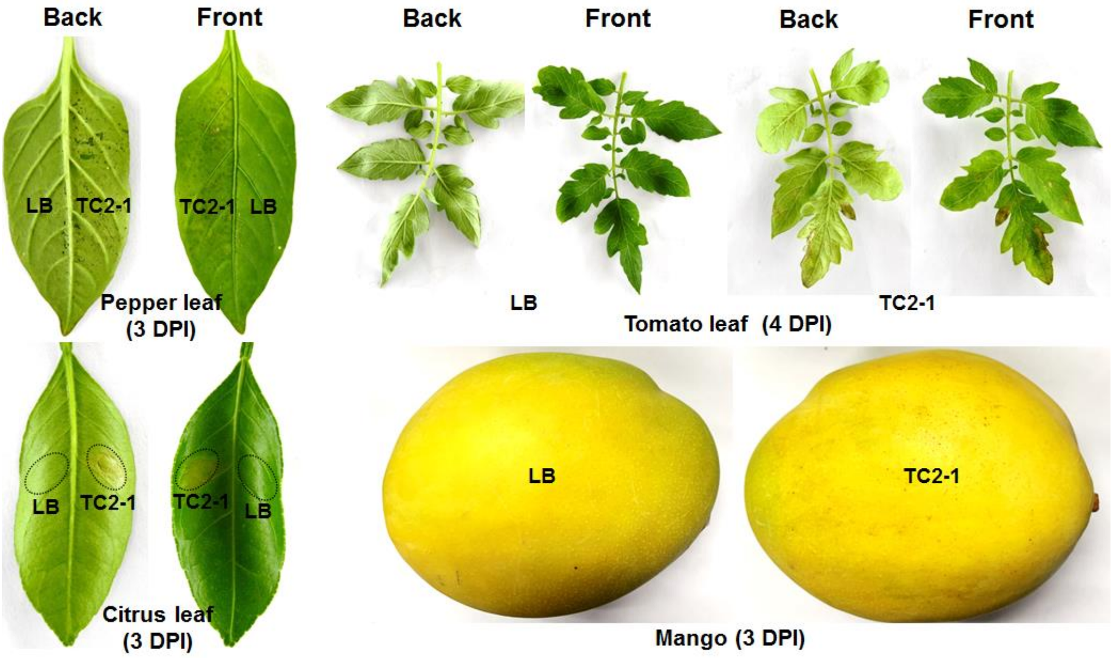

Fig. S6. Pathogenicity tests of TC2-1 on pepper, citrus and tomato leaves, and mango fruit. LB medium was used as negative control.
